# Prediction of Ocular Toxicity of Prostaglandin F_2α_ Analogs Based on Local Computational Models: Superiority over Global Models and Experimental Validation

**DOI:** 10.3390/molecules31173116

**Published:** 2026-09-05

**Authors:** Xinyi Lu, Liping Ren, Chen Wang, Haiming Liao, Lisha Liu, Miaomiao Han, Lanying He, Dousheng Zhang, Huihong Fan, Mingzhe Xu

**Affiliations:** 1 School of Pharmaceutical Engineering, Shenyang Pharmaceutical University, Shenyang 110016, China; xyyx_lu@163.com; 2 National Institutes for Drug Control, Beijing 102629, China; renliping@nifdc.org.cn (L.R.); wangchen@nifdc.org.cn (C.W.);

**Keywords:** ocular toxicity, prediction, computational models, PGF2α, latanoprost, virtual molecular docking, SIRT3

## Abstract

Drug-induced ocular toxicity is difficult to predict and evaluate, particularly for prostaglandin F2α (PGF2α) analogs used in the management of glaucoma. Traditional global predictive models, which are built with large and various datasets (*n* = 6187), provide systematic false-negative results for groups of structurally uniform compounds. To address this limitation, we developed special local classification models for PGF2α analogs. A structurally consistent local training dataset (*n* = 350) was assembled using Murcko scaffold filtering and Tanimoto similarity selection (≥0.5). Binary classification models were built using three different molecular fingerprints in combination with machine learning and two deep learning methods. Although two-dimensional molecular representations do not encode stereochemistry, computational predictions still apply to the shared two-dimensional scaffold of these compounds. These improved models were used to predict the ocular toxicity of latanoprost and its related impurities. The local models (*n* = 350) provided accurate toxicity predictions for latanoprost, latanoprost acid, 15(S)-latanoprost, and the *trans*-5,6-latanoprost isomer, whereas all 16 global computational models provided false-negative results. The experimental assessment performed with primary rabbit corneal epithelial cells (pRCECs) and a human corneal epithelial cell line (HCE-T) confirmed the cytotoxic effects, which were in agreement with the predictions made by the models. Among the tested compounds, 15(S)-latanoprost exhibited the highest cytotoxicity (IC50 = 86.22 μM, 95% CI: 82.90–89.56 μM), followed by *trans*-5,6-latanoprost (IC50 = 106.70 μM, 95% CI: 103.4–110.0 μM) and latanoprost (IC50 = 112.60 μM, 95% CI: 106.7–118.6 μM). At the standard therapeutic dosage (0.005%), no significant toxic response was observed. Virtual molecular docking was employed to explore the mechanism, and all analogs docked favorably into the quinone-binding channel of mitochondrial complex I and the catalytic cleft of SIRT3. These results show how a localized modeling approach is better able to capture structure–toxicity correlations among chemically similar compounds and highlight the critical need for rigorous impurity management in latanoprost products, particularly regarding 15(S)-latanoprost.

## 1. Introduction

The eyes are important sensory organs involved in vision. They have complicated anatomical designs and a special physiological milieu; thus, the eyes are especially susceptible to damage and irritation from external chemical agents [1]. Although the blood–ocular barrier provides defense, some drugs administered systemically or applied locally can still induce toxic effects on ocular tissues. Ocular toxicity from drugs has a wide range of clinical manifestations, such as glaucoma, cataract formation, conjunctival inflammation, corneal inflammation, retinal toxicity, including pigmentary changes, maculopathy, and macular edema [2,3]. It can affect both the anterior and posterior segments of the eye. Adverse ocular reactions induced by pharmaceuticals often develop gradually and may only be discovered when the clinical application has been widely performed, making early prediction and risk evaluation difficult. This trend indicates the need to develop predictive approaches in this therapeutic domain [4,5,6].

**Figure 1:**
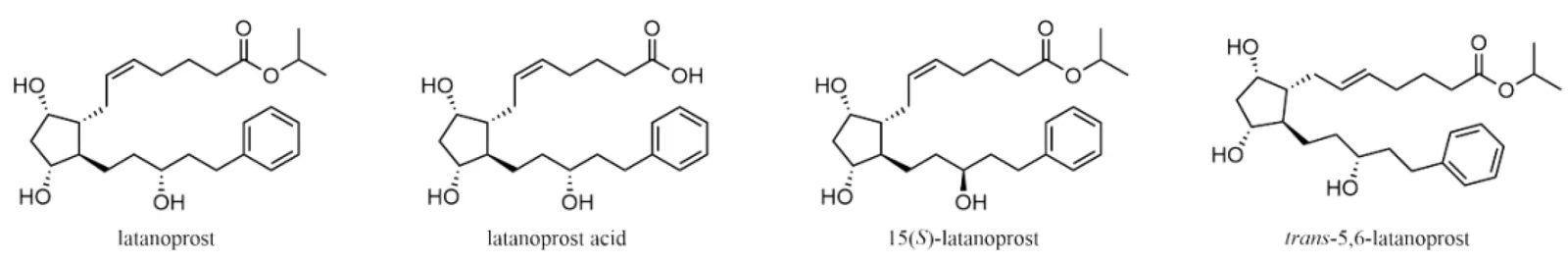
Structures of latanoprost, latanoprost acid, 15(*S*)-latanoprost and *trans*-5,6-latanoprost.

Prostaglandin F2α (PGF2α) analogs decrease intraocular pressure by stimulating prostaglandin FP receptors present in different structures of the eye. These receptors are mainly present in the corneal epithelium, ciliary epithelium, muscle, and iris stroma [7]. Latanoprost was the first PGF2α analog approved by the U.S. Food and Drug Administration (FDA) in 1996. Other compounds such as bimatoprost, travoprost, and tafluprost have also been introduced [8]. These agents are mostly used as first-line treatments for glaucoma; however, adverse ocular reactions related to latanoprost have been reported [9]. The assessment of the safety profile of latanoprost eye drop formulations has mostly focused on preservatives and inactive ingredients [10,11,12]. The synthesis of latanoprost can give rise to important impurities, such as geometric isomers and diastereomers, because of the presence of chiral centers in the latanoprost molecular structure [8,13,14] (Figure 1). However, the possible toxic effects of these impurities on ocular tissues have not been studied.

In 1944, Draize et al. developed a rabbit eye model to test potential chemical substances that irritate the eyes [15]. In 1961, the Draize test was recognized by the FDA and included in the safety assessment procedures for food, drugs, and cosmetic products [16]. Later, the Organisation for Economic Co-operation and Development (OECD) adopted it as a standard procedure for testing the eye irritation potential of chemicals [17]. Problems with the method, such as not always obtaining the same results, ethical concerns (in agreement with the 3Rs principles, replacement, reduction, and refinement), and anatomical and physiological differences between the rabbit and human eyes, have led to the search for alternatives to animal testing. Alternatives include ex vivo organ culture systems, in vitro assays with cell cultures, and models based on reconstructed human corneal epithelial tissues [18].

Current computational methods for predicting ocular toxicity can be divided into three categories: models based on physicochemical descriptors, approaches that combine data-driven machine learning, and architectures that use deep learning for representation. Examples include the QSAR-21 expert system [19], multitask CapsNet deep learning models introduced by Wang et al. [20], and the STopTox platform [21]. However, most of these techniques are based on global modeling (i.e., models trained on datasets containing structurally diverse compounds that span a broad chemical space). The methodology is based on the idea that the greater the chemical variety, the better the applicability of the model to different situations [22]. This principle is true for libraries of compounds with large structural differences; however, it can lead to significant interference when dealing with specific groups of compounds confined to a specific chemical space [23]. The other is local structure–toxicity relationship (STR) modeling (also called embedded cluster modeling (ECM)), which was initiated by Cronin and associates [23], and provides another complementary approach that focuses on structurally uniform groups with identical core structures or operating mechanisms. This method allows for the extraction of more relevant structure–activity correlations, and by construction, it limits the predictions to the interpolation range of the training dataset [24,25]. Although local modeling has some advantages and has been used for many other toxicological endpoints, its systematic application to the prediction of the ocular toxicity of PGF2α analogs has not yet been reported. These molecules are ideal candidates for local modeling because they share a prostanoic acid core structure with multiple stereocenters. Thus, their chemical domains are structurally uniform but stereochemically complex. In addition, their main impurities, such as 15(S)-latanoprost, *trans*-5,6-latanoprost, and latanoprost acid, differ from the parent compound only in stereochemical configuration or in the hydrolysis state of the isopropyl ester (free carboxylic acid) [13,14]. This is a subtle and complex problem for prediction.

Therefore, the aims of this study were as follows: (1) to build specific local computational models for the PGF2α analogs by gathering a training dataset that is as structurally similar as possible and comparing the performance of the local models built from different sources of data with the global models; (2) to use the improved local models to predict the ocular toxicity profiles of latanoprost and its main impurities, since there are no comprehensive safety assessments of the ocular use of these molecules; (3) to experimentally corroborate the model predictions with in vitro assays on corneal epithelial cells using primary rabbit corneal epithelial cells (pRCECs) in a short exposure test and human corneal epithelial cells (HCE-T) in a continuous (24 h) exposure experiment.

## 2. Results

### 2.1. Ocular Toxicity Prediction Models for PGF_2α_ Analogs

#### 2.1.1. Optimal Hyperparameter Combinations

The best hyperparameter configurations for each ocular toxicity prediction model, as determined by 10-fold cross-validation, are presented in Table 1.

In Table 1, we compile the best-performing hyperparameter settings for each of the 16 machine learning (ML) models considered in this study. These include k-nearest neighbors (KNN), support vector machine (SVM), random forest (RF), extreme gradient boosting (XGBoost), convolutional neural network (CNN), and graph convolutional network (GCN). KNN, SVM, RF, XGBoost, and CNN were combined with RDKit, MACCS, and Morgan fingerprint representations. To determine the best parameters, we performed a hyperparameter optimization procedure using 10-fold cross-validation on the training dataset. The final chosen parameters varied significantly between different algorithmic approaches and fingerprint categories, which showed the different structural assumptions intrinsic to the design of the model. For the RF implementations, the optimal number of estimators ranged from 300 to 500, and the maximum tree depth ranged from none (no limit) to 10. For the XGBoost configurations, the best learning rates were comparably lower, ranging from 0.05 to 0.1, and were in combination with more constrained maximum tree depths of 2 to 6. For CNN-based architectures, the kernel size (16 or 32) and learning rate (0.0005–0.001) were optimized as tunable hyperparameters, while the remaining architectural settings were fixed: 64 convolutional filters, 128 units in the dense layer, and a dropout rate of 0.3. The GCN model employed three graph convolutional layers with a hidden dimension of 64, a dropout rate of 0.2, and a learning rate of 0.005. Subsequently, a global mean pooling operation and a final dense layer were used for the output. The optimization was conducted in two stages: an initial coarse grid search followed by a refinement search around the best-performing configurations. Section 4.2.5 reports the complete search space covered across both stages.

#### 2.1.2. Model Performance Evaluation

The external validation set contained 70 compounds, of which only 18 were non-toxic (negative) instances. Thus, there was a significant imbalance between the classes, with a positive-to-negative ratio of approximately 2.9:1. Correspondingly, the smallest possible increase in SP was 1/18 (approximately 5.6%). The correct classification of every negative sample yielded a perfect SP value of 1.0000, regardless of how the positive samples were classified. This low granularity in the measure of SP is given by the statistics of having such a small sample, and it does not have to be interpreted as evidence of a robust generalization of the model. The restricted size of the external test set (*n* = 70, including only 18 negatives) is a relevant limitation of the present investigation. All 16 models evaluated in this subsection and in Section 2.1.3, Section 2.1.4, Section 2.1.5 and Section 2.1.6 are local STR models developed on the PGF2α-focused dataset (*n* = 350); the corresponding global models trained on the full dataset (*n* = 6187) are compared separately in Section 3.2.

**Figure 2:**
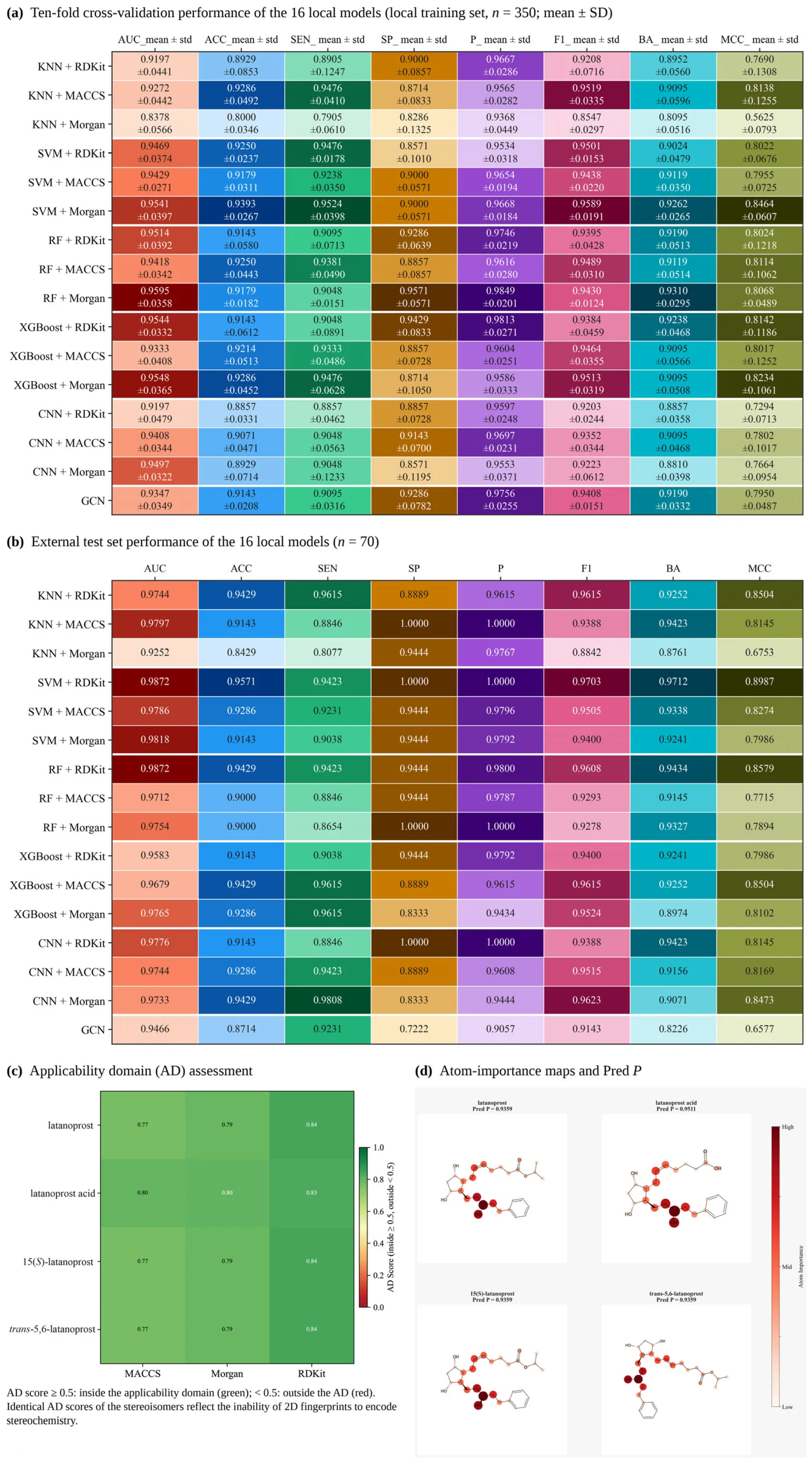
Performance and interpretation of the 16 local models for predicting the ocular toxicity of PGF_2α_ analogs. (**a**) Ten-fold cross-validation performance of the 16 local models on the local training set (*n* = 350; mean ± SD); (**b**) performance of the 16 local models on the external test set (*n* = 70); (**c**) applicability domain (AD) assessment of the four test compounds based on MACCS, Morgan, and RDKit fingerprints (AD score ≥0.5 indicates that the compound lies inside the AD); (**d**) atom-importance maps and predicted toxicity probabilities (Pred P) of the four test compounds. AUC, area under the ROC curve; ACC, accuracy; SEN, sensitivity; SP, specificity; P, precision; F1, F1-score; BA, balanced accuracy; MCC, Matthews correlation coefficient. Latanoprost, 15(S)-latanoprost, and *trans*-5,6-latanoprost share identical 2D structural representations and therefore receive identical AD scores and Pred *p* values.

The model performance was evaluated separately using 10-fold cross-validation and on the independent external test set (Table 2 and Table 3, respectively; all metrics are reported with 95% confidence intervals). Under 10-fold cross-validation, the top three models by AUC were RF + Morgan, RF + RDKit, and XGBoost + RDKit (Figure 2a). Among these, RF + RDKit achieved the highest specificity (SP = 0.9714, 95% CI: 0.9068–1.0000) and precision (*p* = 0.9909, 95% CI: 0.9703–1.0000), along with a balanced accuracy (BA) of 0.9405 (95% CI: 0.8942–0.9868). We note that the cross-validation negative class contained only 70 compounds; consequently, the specificity estimate, while numerically high, carries a wide confidence interval and should be interpreted with caution rather than as a precise measure of the model’s true-negative performance.

On the external test set, the top three models by AUC were RF + RDKit, RF + Morgan, and SVM + RDKit (Figure 2b). The highest sensitivity on the external set was achieved jointly by SVM + Morgan (SEN = 0.9808, 95% CI: [0.9423, 1.0000]) and RF + Morgan (SEN = 0.9808, 95% CI: [0.8036, 1.0000]). Full performance metrics with 95% confidence intervals for all 16 models under both evaluation schemes are provided in Table 2 (cross-validation) and Table 3 (external test set).

#### 2.1.3. Y-Randomization Validation

To avoid the fact that the observed predictive results were due to random correlations, Y-randomization (also called permutation) tests were performed for the best models. For 100 independent randomization iterations, the labels for the endpoint (toxic versus non-toxic) were randomly shuffled, and the original feature matrices were unchanged. In each cycle, the models were retrained and assessed in the same way with the same hyperparameter configuration and 10-fold cross-validation procedure. The performance of the Y-randomized models was dramatically reduced in all evaluation measures (average randomized AUC was below 0.6, and average randomized BA was below 0.55). This result confirms that the predictive power of the original models did not arise from a random association or an intrinsic bias in the dataset. For the three leading models (RF + RDKit, RF + Morgan, and XGBoost + RDKit), the calculated Z-scores were greater than 3.5 (corresponding to a *p* value less than 0.001), providing strong statistical support that their performance levels were significantly higher than those obtained with randomly permuted data.

#### 2.1.4. Statistical Comparison of Model Performances

The statistical significance of the performance variation between the best models was tested using a pairwise comparison. DeLong’s test was used to test the differences in the AUC, and McNemar’s test was used to test the differences in accuracy in the external validation dataset. The AUC value for the Random Forest model combined with RDKit descriptors (0.9861) was not significantly different from that of the random forest model using Morgan fingerprints (0.9861, *p* = 0.512) or the SVM model with RDKit descriptors (0.9861, *p* = 0.287), according to the results of DeLong’s test. This indicates that the three best models have similar discrimination abilities. However, the RF + RDKit model showed borderline significant superiority compared to the convolutional neural network model using Morgan fingerprints (0.9814, *p* = 0.048). In the external validation set, the overall classification accuracy did not differ significantly according to McNemar’s test when comparing the RF + RDKit combination with RF + Morgan (*p* = 0.774), XGBoost + RDKit (*p* = 0.683), or SVM + RDKit (*p* = 0.452). These results confirm the stability of the model performance hierarchy and show that the better efficacy of the RF + RDKit approach is not due to an outlying model but shows authentic predictive information contained in the molecular descriptor sets.

#### 2.1.5. Applicability Domain Assessment Results

We also performed applicability domain (AD) evaluations for all 16 local models to determine the reliability of the prediction for the four target PGF2α analogs. For every combination of the compounds and the 15 fingerprint-based models, the AD scores were >0.5. This indicates that latanoprost, latanoprost acid, 15(S)-latanoprost, and *trans*-5,6-latanoprost were structurally included in the training data for each model, as shown in Figure 2c. In particular, all four substances satisfy the graph convolutional network (GCN) AD criterion, which is evaluated using the Euclidean distance to the 5-nearest neighbors in the graph-embedding space (Table 4). This implies that the predictions are chemically reasonable and not due to extrapolation errors. The applicability domain of the GCN is based solely on two-dimensional structural similarity. Here, we do not consider the variation in stereochemistry; this is discussed in more detail in Section 2.1.7.

#### 2.1.6. Prediction Results

Sixteen local STR models, all developed on the PGF2α-focused dataset (*n* = 350), were used to evaluate the ocular toxicity potential of the four chemical compounds, and the results are presented in Table 5. For latanoprost, 15(S)-latanoprost, and *trans*-5,6-latanoprost, 13 predictive tools indicated toxic classification. In the case of latanoprost acid, all 16 models indicated toxicity. Thus, each of the four substances was classified as an ocular toxicant by the large majority of the models (13/16 for latanoprost, 15(S)-latanoprost, and *trans*-5,6-latanoprost; 16/16 for latanoprost acid). The strong concordance between the different models provides good computational support for the ocular hazards of these compounds.

The fact that the prediction scores are consistently high (keeping the values above 0.9 for the positive results in the case of the random forest, XGBoost and convolutional neural network) further corroborates the trustworthiness of the toxicity assessments. The excellent agreement of the predictions from a huge array of algorithmic frameworks (from distance-based algorithms such as K-nearest neighbors, margin-based algorithms such as SVM, tree-based ensembles such as random forest and XGBoost, deep learning methods such as CNNs, and graph-based algorithms such as graph convolutional networks) is further proof that the molecular structural features of the PGF2α analogs have a strong correlation with the ocular adverse effects in the training data.

#### 2.1.7. Model Interpretation and Stereochemistry Limitation

Gradient saliency analysis performed on the GCN model revealed a meaningful relationship between structural features and toxicity (Figure 2d). The locations with high gradient values, which indicate molecular characteristics that would strongly affect the prediction of toxicity, are as follows: (i) the hydroxyl groups attached to the Rω side chain and the adjacent carbon atoms; (ii) the double bond together with the carboxylic acid or ester functional groups on the Rα side chain; and (iii) the polar functional groups attached to the prostaglandin core structure. The benzene ring system had a low saliency score, indicating that it played a weak role in the model’s toxicity assessment.

The GCN framework provided the same forecasted probability of 0.9956 for latanoprost, 15(S)-latanoprost, and *trans*-5,6-latanoprost in the test set, although these compounds had different stereochemical configurations at the C15 carbon and C5-C6 double bond. A slightly reduced probability of 0.9918 was obtained for the latanoprost acid. A significant drawback of the model is that it provides the same predictions for the three stereoisomeric forms of latanoprost. The underlying GCN structure depends only on two-dimensional molecular graphs, which cannot encode specific stereochemical information, such as E/Z isomerism or R/S chirality. It does not distinguish between the stereoisomers. In the case of PGF2α analogs, this is very important because the stereochemistry of these compounds is, in principle, the governing factor for their biological function, affinity for target receptors, and pharmacological specificity. The failure of the GCN model to recognize stereochemical differences could explain why the given substances have the same likelihood of being toxic; in living organisms, they could have different toxicities. This highlights the importance of graph neural network approaches that consider stereochemical awareness.

However, further investigation indicated that this limitation is not specific to graph-based architecture but originates from the composition of the training data used across the entire modeling pipeline. Latanoprost, 15(S)-latanoprost, and *trans*-5,6-latanoprost were identical inputs to all 15 fingerprint-based models as well. The identical toxicity classifications assigned to the three stereoisomers (Table 5) arise from the two-dimensional nature of the entire modeling pipeline, and the predictions should be interpreted as characterizing the shared two-dimensional scaffold rather than individual stereoisomers. Direct inspection of the stereochemical content of both training datasets further showed that explicitly defined stereocenters were extremely scarce. The local dataset (*n* = 350) contained no compounds with assigned stereocenters, and the global dataset (*n* = 6187) contained only two such compounds (0.03%). Consequently, even if chirality-aware encodings (e.g., Morgan fingerprints generated with the chirality flag enabled) are applied, the chirality-sensitive bits are constant across the training samples and therefore non-informative for supervised learning.

Addressing this limitation will require substantially expanding the training data to include a larger and more balanced representation of stereochemically annotated compounds, and, where feasible, curating compounds with unassigned but structurally chiral centers against source literature or database records to recover their defined configurations.

### 2.2. In Vitro Corneal Cytotoxicity Validation

#### 2.2.1. Correlation Between In Silico Predictions and In Vitro Toxicity

The biological relevance of the computational toxicity forecasts for the four PGF2α analogs, which were predicted to be toxic by almost all (13 out of 16 or 16 out of 16) models, was verified by comparing them with experimental cytotoxicity. In vitro, each of the four compounds was cytotoxic, depending on its concentration. In agreement with the computer-based forecasts, when applied at the minimal tested concentration of 0.005%, all substances maintained cell viability at approximately 97–105% with no statistically meaningful variations (*ns*, *p* > 0.05), which is in agreement with a non-toxic designation. In contrast, at 0.05% in the pRCEC-STE assay, all four compounds induced a significant decline in cell viability (*** *p* < 0.001); in the 24 h HCE-T assay, the three ester analogs showed concentration-dependent cytotoxicity, whereas latanoprost acid showed no significant toxicity up to 1 mM, together validating the toxic classification expected by almost all computational models. This qualitative agreement between the theoretical predictions and laboratory results shows that localized computational models may be valuable for evaluating the ocular toxicity of PGF2α analogs. However, in silico models provide binary classifications of toxicity (toxic or non-toxic) and cannot predict experimentally observed concentration-dependent cytotoxicity levels. It should also be emphasized that the binary model labels denote toxicological hazard potential rather than exposure-adjusted risk: the experimental ground truth supporting the toxic classification corresponds to the elevated concentrations (≥0.05%), whereas no toxicity was observed at the therapeutic concentration (0.005%).

#### 2.2.2. Short Time Exposure on pRCECs

**Figure 3:**
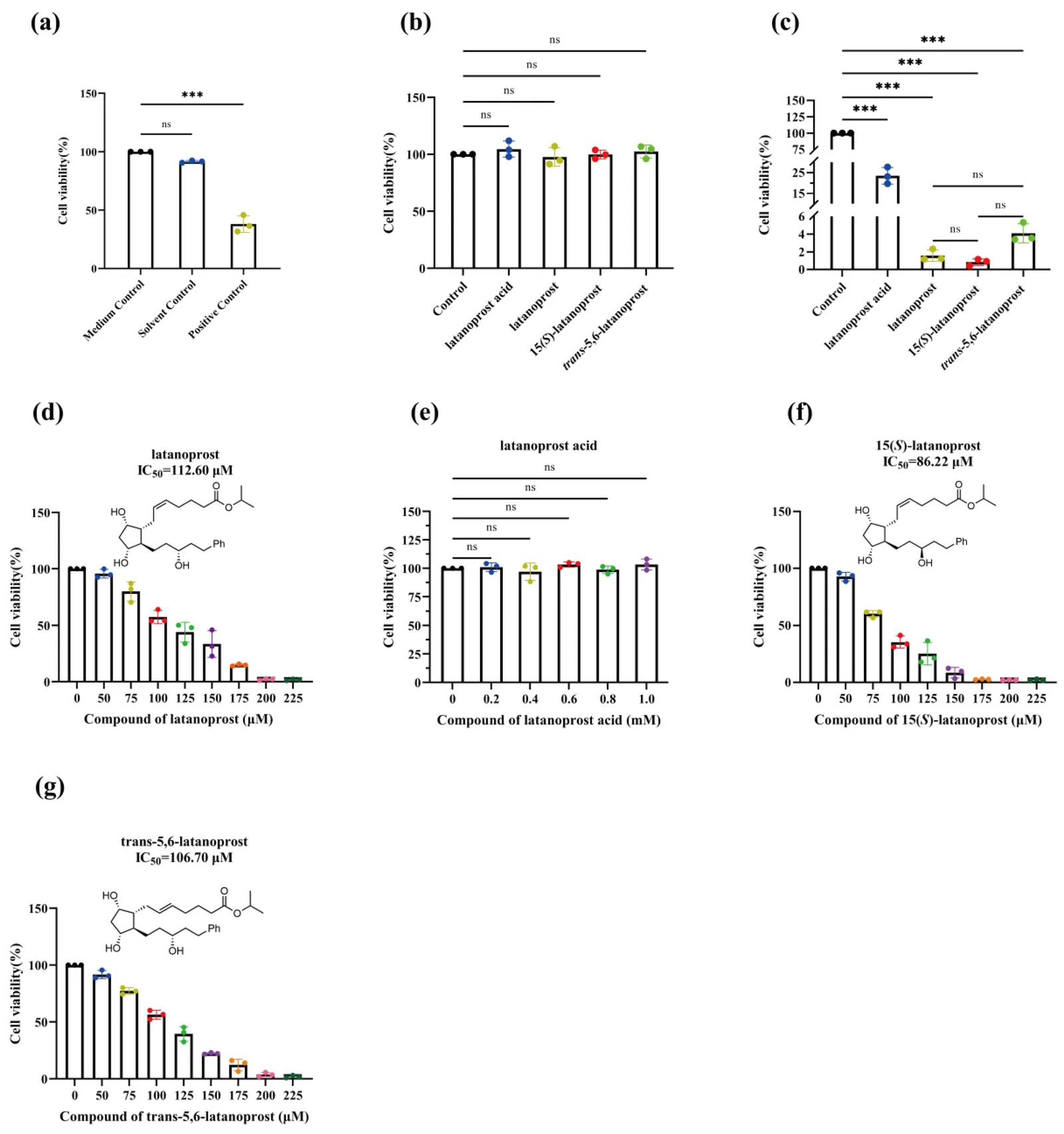
Toxicity evaluation of latanoprost and its impurities in ocular cell models. (**a**) Cell viability of pRCEC control groups, including culture medium control, solvent control (5% DMSO/saline), and positive control (0.01% SLS). Cell viability of pRCECs following 5 min exposure to latanoprost and its impurities at (**b**) 0.005% and (**c**) 0.05%, assessed by CCK-8 assay. (**d**–**g**) Concentration-dependent cytotoxicity in HCE-T after 24 h treatment, assessed by MTT assay: (**d**) latanoprost at concentrations of 50, 75, 100, 125, 150, 175, 200, and 225 µM; (**e**) latanoprost acid at concentrations of 0.2, 0.4, 0.6, 0.8, and 1.0 mM; (**f**) 15(*S*)-latanoprost at concentrations of 50, 75, 100, 125, 150, 175, 200, and 225 µM; (**g**) *trans*-5,6-latanoprost at concentrations of 50, 75, 100, 125, 150, 175, 200, and 225 µM. Data are presented as mean ± SD (*n* = 3). Statistical significance was determined using one-way analysis of variance (ANOVA). Asterisks indicate significant differences compared with the control (*** *p* < 0.001); ns, not significant (*p* > 0.05).

Assay validity was verified using control groups run in parallel (Figure 3a). Cell viability in the solvent control group (5% dimethyl sulfoxide (DMSO)/saline) was 91.55% ± 0.98% relative to the culture medium control, with no statistically significant difference between the two groups (*ns*, *p* > 0.05), and the solvent control viability exceeded the 80% acceptance threshold in every independent run, indicating that 5% DMSO exerted no appreciable cytotoxic effect on pRCECs under the 5 min exposure conditions. Treatment with the positive control, 0.01% sodium lauryl sulfate (SLS), reduced cell viability to 38.00 ± 7.14%, below the 50% threshold defined for assay validity, confirming that the pRCEC-STE system retained sufficient sensitivity to detect a known irritant.

We performed an acute cytotoxicity evaluation of primary rabbit corneal epithelial cells (pRCECs) using a short-term exposure (STE) test at concentrations of 0.005% and 0.05%. At a concentration of 0.005%, all four tested substances exhibited cell viability rates of approximately 97–105%. We did not observe any statistically significant variations when comparing any compound to the vehicle control (*ns*, *p* > 0.05). The results shown in Figure 3b indicate that no acute cytotoxic effects were observed at this concentration (0.005%).

At a concentration of 0.05%, we observed a significant decrease in cell viability for each of the four substances (*** *p* < 0.001 vs. vehicle). The percentages recorded for cell survival were as follows: latanoprost acid, 23.45% ± 4.02%; latanoprost, 1.57% ± 0.65%; 15(S)-latanoprost, 0.82% ± 0.37%; and *trans*-5,6-latanoprost, 4.09% ± 1.07%. We want to remark that latanoprost acid showed a notably higher residual viability (23.45%) in comparison with the other three analogs, which in all cases are below a 5% survival rate. This suggests that the free acid form may induce a different cytotoxic mechanism or exhibit a different pattern of cell absorption compared to the isopropyl ester derivative and its stereoisomeric forms, as shown in Figure 3c.

The STE protocol, originally developed and validated with SIRC cells [26] and adopted as an OECD Test Guideline 491 [27], was adapted to pRCECs because primary rabbit corneal epithelial cells more closely reproduce the in vivo corneal response to irritants than immortalized cell lines [28,29]. This adaptation is acknowledged as a limitation of this study. The final DMSO concentration of 5% (*v*/*v*) during the 5 min exposure was relatively high; its intrinsic effect was therefore documented directly: the matched solvent control retained 91.55 ± 0.98% viability relative to the medium control (*ns*, *p* > 0.05, above the 80% acceptance threshold), and all compound viability data were normalized to this vehicle control [30].

#### 2.2.3. Continuous Exposure Assay in HCE-T Cells

The half-maximal inhibitory concentration (IC_50_) was measured to evaluate the cytotoxic effects of sustained exposure using the HCE-T human corneal epithelial cell model. HCE-T cells were exposed for 24 h to 50, 75, 100, 125, 150, 175, 200, and 225 μM of latanoprost, 15(S)-isomer, and the *trans*-5,6 derivative, respectively. All three agents showed a clear, dose-dependent reduction in cell viability. The data were fitted using nonlinear regression, and the IC_50_ estimates obtained were 112.60 μM (95% CI: 106.7–118.6 μM), 86.22 μM (95% CI: 82.90–89.56 μM), and 106.70 μM (95% CI: 103.4–110.0 μM) for latanoprost, 15(S)-latanoprost, and *trans*-5,6-latanoprost, respectively. Among the three compounds for which an IC_50_ was determined, 15(S)-latanoprost showed the highest cytotoxic potency, as indicated by the lowest IC_50_. The IC50 values of latanoprost and *trans*-5,6-latanoprost were nearly identical, indicating similar cytotoxic activities, as shown in Figure 3d,f,g. In contrast, when HCE-T cells were treated with latanoprost acid at concentrations of 0.2, 0.4, 0.6, 0.8, and 1.0 mM, no significant change in cell survival was observed compared to the untreated control group at any concentration tested. As shown in Figure 3e, there was no statistically significant difference in cell viability at the tested concentrations (*ns*, *p* > 0.05). The relative cytotoxicity ranking obtained from the continuous exposure assay showed that 15(S)-latanoprost had the greatest potency, followed by *trans*-5,6-latanoprost and latanoprost, which had similar effects, and latanoprost acid, which had the least potency. This study provides quantitative data on the different toxicological profiles of structurally similar PGF2α compounds. This result is of great importance for setting acceptable impurity thresholds, as 15(S)-latanoprost, a frequently encountered stereoisomeric contaminant of latanoprost pharmaceutical preparations, showed the most pronounced cytotoxic effect of all the substances tested.

### 2.3. Virtual Molecular Docking Results

**Figure 4:**
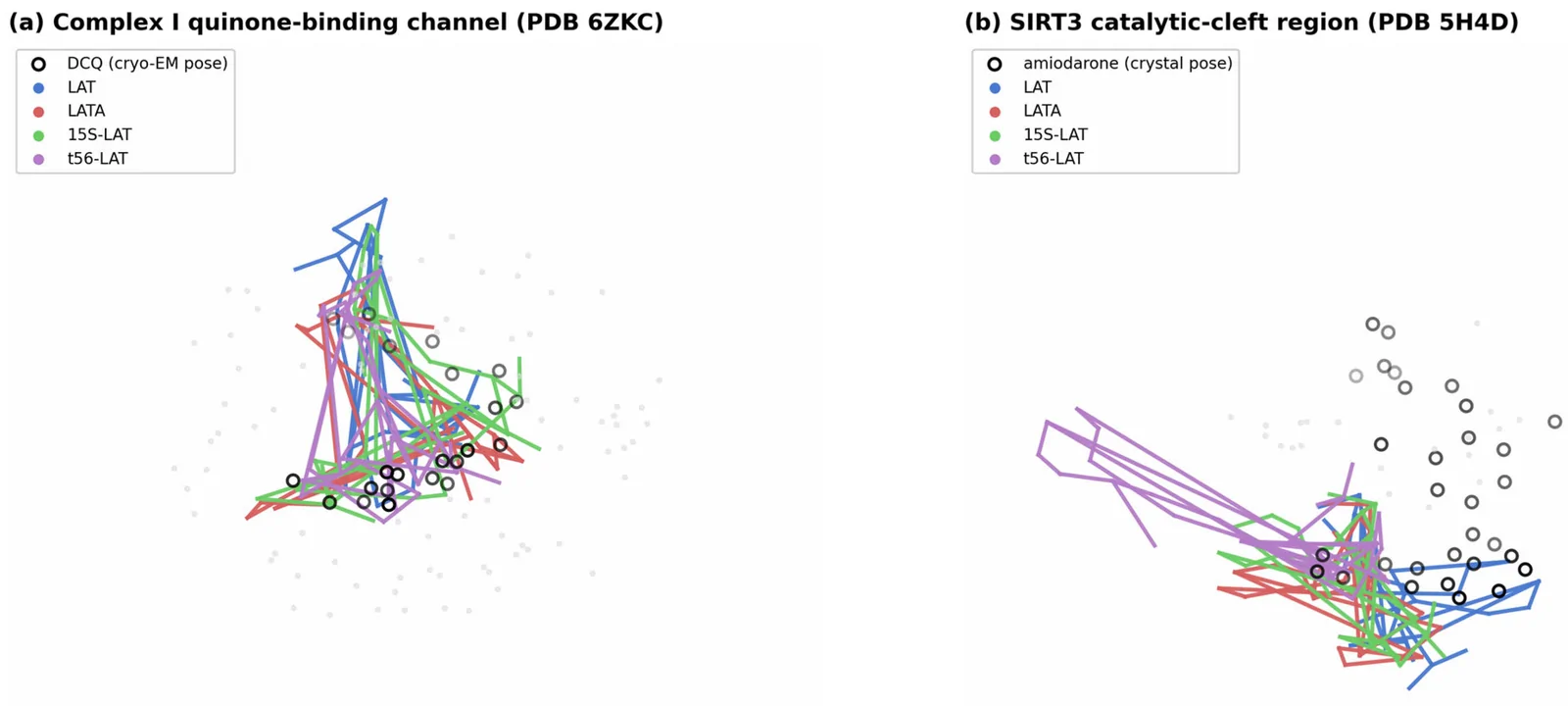
Best-scoring docked poses (sticks) of latanoprost (blue), latanoprost acid (red), 15(S)-latanoprost (green), and *trans*-5,6-latanoprost (purple) in (**a**) the quinone-binding channel of mitochondrial complex I (PDB 6ZKC) and (**b**) the catalytic-cleft region of SIRT3 (PDB 5H4D). Binding-site residues are shown as grey spheres; open black circles mark the crystallographic ligand positions (decylubiquinone and amiodarone, respectively).

**Figure 5:**
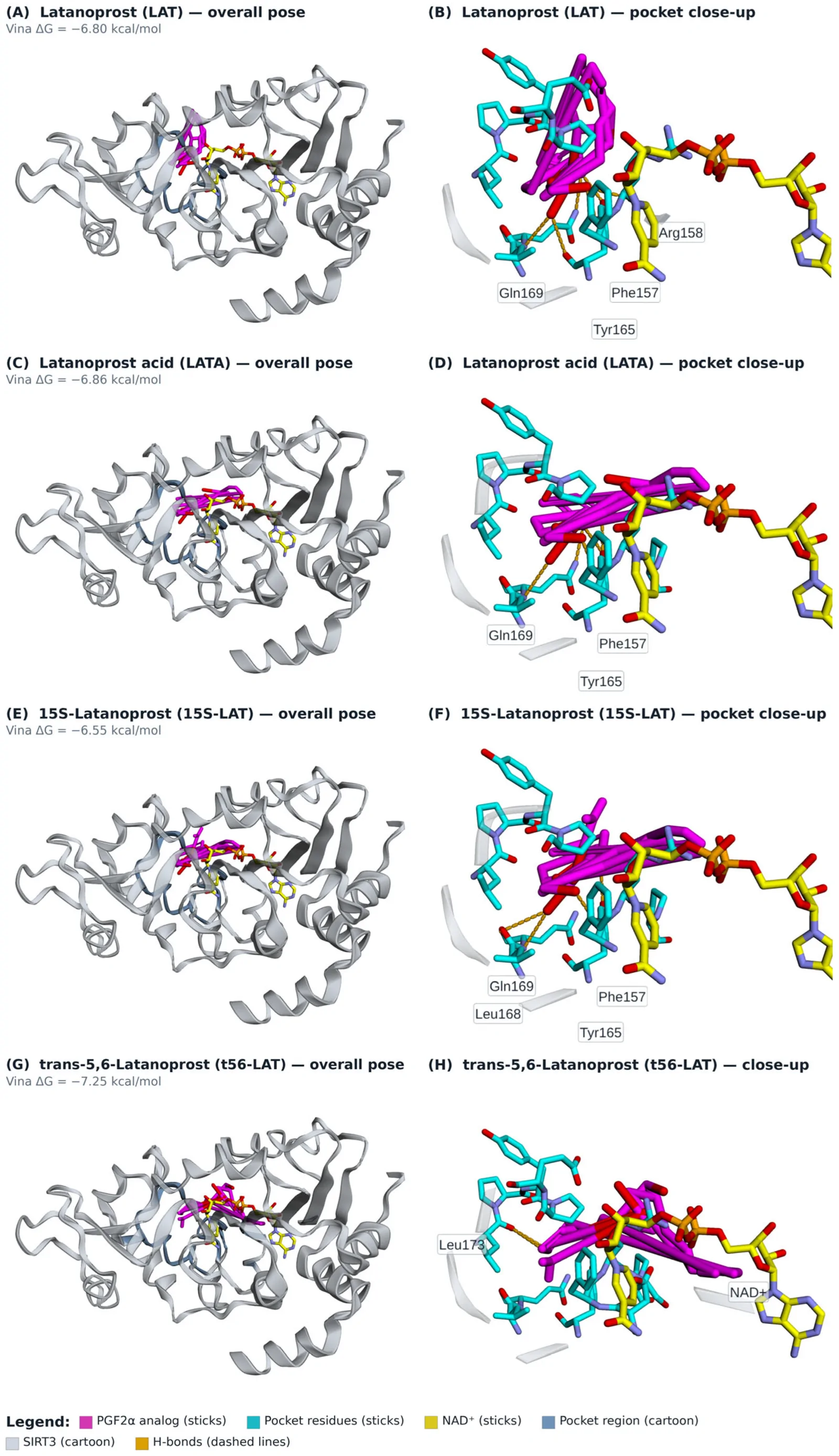
Binding pockets of the four PGF2α analogs docked into SIRT3 (PDB 5H4D, Heteroatoms are colored by element: oxygen in red, nitrogen in blue). (**A**,**C**,**E**,**G**) Overall views: SIRT3 is shown as a light-grey cartoon, with the pocket-lining residues as cyan sticks, NAD^+^ as yellow sticks, and the ligand as magenta sticks; the bound ligand position is highlighted within the pocket region. (**B**,**D**,**F**,**H**) Close-up views of the binding pocket: interacting residues are shown as cyan sticks and labeled; hydrogen bonds (donor–acceptor heavy-atom distance <3.6 Å) are indicated by gold dashed lines. (**A**,**B**) Latanoprost; (**C**,**D**) latanoprost acid; (**E**,**F**) 15(S)-latanoprost; (**G**,**H**) *trans*-5,6-latanoprost. Best-pose AutoDock Vina docking scores are indicated in each panel row.

To explore the mechanistic basis of the cytotoxicity observed in the corneal cell models, latanoprost and its impurities were subjected to ligand-based target prediction and molecular docking. ProTox-3.0 [31] returned a single positive signal across its 27 models: the mitochondrial complex I molecular initiating event (MIE) “NADH–quinone oxidoreductase” was predicted to be active for the three PGF2α analogs (probability 0.50) but predicted inactive for latanoprost acid (probability 0.56 for the inactive class, Table S3). This prediction is consistent with in vitro data, in which latanoprost acid was far less cytotoxic than the three analogs in both the pRCEC-STE assay (23.45% vs. <5% residual viability at 0.05%) and the HCE-T assay (no toxicity up to 1 mM). SwissTargetPrediction yielded a corroborating target profile (Table S2) [32]. Within the adverse outcome pathway framework [33], this ester-specific signal nominated mitochondrial complex I and its upstream regulator SIRT3 [34,35] for docking studies. Redocking of decylubiquinone into complex I (PDB 6ZKC) reproduced the cryo-EM pose (RMSD 2.02 Å), validating the protocol. All four analogs docked into the quinone-binding channel with scores of −9.51 to −10.55 kcal/mol, which were well below that of the redocked substrate analog (−7.16 kcal/mol), overlapping the crystallographic quinone position (Figure 4a) and recapitulating the canonical quinone-site anchors on NDUFS2 and NDUFS7 [36,37] (Table S4; Figure S4). In SIRT3 (PDB 5H4D), the four analogs occupied a hydrophobic groove adjacent to the bound NAD^+^ (Figure 4b), with scores of −6.54 to −7.25 kcal/mol (Table S5), which were comparable to or better than the redocked amiodarone (−6.02 kcal/mol). The binding pockets of the four complexes are shown in Figure 5. Latanoprost, latanoprost acid, and 15(S)-latanoprost were anchored by hydrogen bonds to pocket-lining residues (Phe157, Tyr165, Gln169, and Arg158 or Leu168), whereas the strongest binder, *trans*-5,6-latanoprost, relied mainly on hydrophobic contacts with a direct contact with NAD^+^. These results support the physical plausibility of a mitochondrial mechanism, either direct interference with complex I or indirect disruption via SIRT3-mediated destabilization of complex I, underlying the concentration-dependent cytotoxicity observed in vitro.

## 3. Discussion

### 3.1. Rationale and Performance of Scaffold-Based Local Modeling

This study is based on the hypothesis that localized STR models built with chemically uniform sets of molecules with specific structures can provide reliable predictions in their particular applicability domains. In contrast, general models built with structure-varied datasets covering wide areas of chemical territory can have predictive restrictions. This hypothesis is based on one of the basic tenets of cheminformatics, which states that molecules with strong structural similarities often exhibit similar biological effects. This tenet has supported the scaffold-centered modeling methodology for over 20 years [25,38]. In this study, a localized model of ocular toxicity of PGF2α analogs was developed. It was trained on the 280-compound training partition (80%) of the local dataset of 350 structurally related prostaglandin derivatives. The best-performing model, RF + RDKit, achieved an AUC of 0.9612, specificity of 0.9714 (95% CI: 0.9068–1.0000), and a balanced accuracy of 0.9405 (95% CI: 0.8942–0.9868) under cross-validation.

The local model was trained and assessed using a dataset with structural homogeneity (350 samples), and the test compounds had structures similar to those of the compounds in the training set. The global models (*n* = 6187) were validated using an external test set (70 samples) with significant structural diversity and large chemical space. Because of the completely different compositions of the test sets, it is not appropriate to compare the performance metrics to claim that one model is “better” than the other model. Regardless of the quality of the models, a model evaluated on compounds structurally close to those in the training data will yield different results than a model tested on a structurally different set of compounds [39].

Rather than stating that our results show that local modeling is generally superior, we suggest a stronger interpretation that for compounds that belong to a well-defined structural class, for example, PGF2α analogs, local models (*n* = 350) built using structurally similar compounds provide reliable predictions in their defined applicability domain. In contrast, global models (*n* = 6187) may find these compounds outside their applicability domain or provide unreliable predictions because of sparse coverage in the training data for that region of the chemical space. Hence, the strength of the proposed model is its reliable performance within the given scope rather than its superiority in all chemical domains. This is an important methodological difference and has immediate consequences for how professionals should use our conclusions when considering the use of local modeling approaches for evaluating pharmaceutical impurities.

We chose a Tanimoto similarity cutoff of 0.5. This is the standard in cheminformatics when searching for structurally similar compounds [40]. This indicates that the molecules generally have sufficient common structural parts to exhibit similar biological effects. We are aware that this cutoff value is not very definitive, and conducting a sensitivity analysis with other values (for instance, 0.4 or 0.6) would increase the robustness of the local modeling approach. Some preliminary investigations suggest that the choice of this parameter results in a trade-off between the comprehensiveness of the model (number of molecules included in the local training dataset) and the structural coherence (degree of resemblance among those molecules).

### 3.2. Comparison of Local and Global Modeling Approaches

In this study, 16 different global STR models (*n* = 6187) were used, employing algorithms such as RF, SVM, KNN, XGBoost, CNN, and GCN. These models used different chemical feature sets, such as Morgan fingerprints, Molecular ACCess System (MACCS) keys, and RDKit fingerprints. For the four prostaglandin F2α analogs (latanoprost, latanoprost acid, 15(S)-latanoprost, and *trans*-5,6-latanoprost), all 16 models predicted non-toxicity. In contrast, the 16 local STR models developed on the PGF2α-focused dataset (*n* = 350) classified all four compounds as toxic (13/16 models for latanoprost, 15(S)-latanoprost, and *trans*-5,6-latanoprost; 16/16 for latanoprost acid; Section 2.1.6 and Table 6). The fact that all of the negative results agree in this huge range of global model architectures points to a systematic bias in this investigation, where the global models seem to be prone to under-predicting the possible ocular toxicity of the prostaglandin analogs.

However, we are careful not to generalize these results. The inference made here is based on the estimation of only four compounds with similar structures, all of which were studied in the same investigation. Therefore, it cannot support a systematic error that is generally valid for all STR models. We can say with certainty that, at least under the conditions of this study, the global models could not properly classify these four pharmacologically important substances.

This perspective is also supported by a comparison with the commonly used general-purpose platform for ADMET forecasting, called toxCSM [41]. As shown in Table 6, toxCSM assigned toxicity scores of 0.0–0.01 to all four compounds for both the eye corrosion and eye irritation endpoints, i.e., confident non-toxic predictions.

We do not imply that toxCSM is inadequate as a versatile framework. Instead, it shows that models designed for general application to various chemical domains and trained to cover a broad range of chemical structures might lose predictive precision for molecules at the periphery of the molecular configuration for which they are used, that is, in regions where the amount of training data is scarce [42]. Models that are designed and trained locally for well-characterized groups of compounds could be another or complementary methodology for quality control in pharmaceutical application. This is not a criticism of a general or broadly applicable platform. This is a call for diverse modeling methodologies: various predictive problems might be better solved with different modeling philosophies, and professionals know that there are compromises in a given strategy [43].

### 3.3. Graph Convolutional Network Analysis and Interpretability Considerations

The GCN model used in this study is a good example of the fine interpretation of the predictions of machine learning models in the field of cheminformatics. The GCN identified all four PGF2α analogs as ocular toxicants, confirming the binary classifications performed by the local model. However, the model assigned the same predicted probability (0.9956) to the stereoisomers latanoprost, 15(S)-latanoprost, and *trans*-5,6-latanoprost, and a slightly lower probability (0.9918) to latanoprost acid. At first glance, this may appear to question the GCN’s trustworthiness. If the model does not differentiate between stereoisomers, can we trust the predictive outcomes of the model?

The solution to this apparent contradiction is to consider classification and ranking as two separate predictive goals [44]. The assignment of the “toxic” label to all four compounds by the GCN is consistent with the presence of ocular toxicity. Toxicity was experimentally confirmed for all four compounds, indicating that the binary prediction was accurate. However, the fact that the probability scores are all the same (0.9956) shows that the model is not able to discriminate the stereoisomers in terms of their strength, which is a task that is different from a simple binary classification. These two facts are not contradictory but are in agreement: the GCN is reliable for determining toxicity but not for ordering chiral variants by their strength.

This differentiation has important consequences for the analysis of molecular explanation maps produced by the GCN. The generated maps pinpointed the elements of the pharmacophore that are linked to ocular toxicity, namely the carboxylic acid/ester terminus of the α-chain with its adjacent double bond, the hydroxyl group of the ω-chain, and the polar substituents of the prostaglandin core (Section 2.1.7). We note that the electrophilic cyclopentenone prostaglandins known to modify cysteine residues through Michael addition [45,46] are structurally distinct from the saturated analogs studied here, which lack this moiety. However, the fact that the probability scores are the same for all four compounds means that the GCN did not discriminate features responsible for the different potencies, such as the stereochemical configuration at the C5-C6 or C15 position. This is a shortcoming of the model design and the nature of the training dataset and not of the insufficiency of the interpretation approach itself [47]. Furthermore, because none of the two-dimensional representations used in this study encoded stereochemistry, the three stereoisomers constitute identical inputs to all 16 local models (not limited to the GCN), yielding identical predictions. Therefore, the computational results characterize their shared two-dimensional scaffold, whereas the experimentally observed potency differences among the stereoisomers provide the stereoisomer-specific resolution that the current models cannot achieve.

### 3.4. Experimental Validation: Convergent Evidence from Complementary Assays

The computational forecasts were verified using two in vitro ocular toxicity tests: the pRCEC short-term exposure (STE) assay and the HCE-T continuous exposure assay.

The pRCEC STE test evaluates the immediate irritation potential of conditions resembling a single topical application and measures cytotoxic damage after a relatively short exposure time of minutes to a few hours [26,27]. In contrast, the HCE-T continuous exposure test evaluates chronic cytotoxic strength by obtaining the IC50 value after prolonged 24 h contact [48]. In previous studies, the negative effects of latanoprost eye drops on the ocular surface were related to the preservative benzalkonium chloride (BAK) and not the drug molecule [49,50]. Inoue et al. [51] reported that using the preservative-free 0.005% latanoprost solution did not cause any significant decrease in the transepithelial electrical resistance or any histomorphological damage in the rabbit cornea. In accordance with these results, latanoprost at a 0.005% concentration did not show any significant cytotoxic effects on pRCECs in the short-term exposure test, indicating that the drug is safe for the ocular surface at the standard therapeutic level. However, when the concentration was increased to 0.05%, all four tested substances resulted in a substantial decrease in pRCEC survival, indicating that each agent could irritate the ocular surface at high concentrations. Situations in clinical practice, such as repeated administration, insufficient dilution of the tear film, or a poor corneal barrier, can lead to a high local concentration. Such conditions may lead to higher drug concentrations at some sites; however, the extent to which they increase the risk of corneal epithelial toxicity in vivo remains unclear and requires further study.

In the 24 h continuous exposure experiment using the HCE-T cell model, latanoprost, its 15(S)-isomer, and the *trans*-5,6-latanoprost variant all showed a clear dose response in cell toxicity. The 15(S)-latanoprost isomer exhibited the most potent cytotoxic effect, as indicated by the lowest half-maximal inhibitory concentration (IC_50_). This result shows that this specific isomer should be strictly monitored during the manufacturing and quality assurance of latanoprost-based pharmaceuticals. When the concentrations exceeded the critical levels, all three tested compounds caused significant damage to the structural and operational states of corneal epithelial cells. Cellular damage can occur through different mechanisms, such as loss of plasma membrane integrity, impairment of mitochondrial activity, and induction of oxidative stress, which are commonly known mechanisms of several cytotoxic substances, as stated in a previous study [52,53]. The molecular mechanism by which prostaglandin F2α analogs exert these toxic effects needs to be investigated in more detail.

One of the important constraints of the HCE-T continuous exposure method is that the culture medium has a buffering capability that could mask the cytotoxicity of latanoprost acid, which was observed in the pRCEC short-term exposure test. This variation underlines the need to use different testing approaches with different exposure parameters and highlights the lack of knowledge regarding which system represents the actual exposure conditions on the ocular surface in a living organism. In this procedure, the substances to be tested were mixed in the culture medium before being added to the cells for analysis. The HCE-T growth medium is composed of buffering salts, amino acids, glucose, and serum proteins that maintain a constant pH of 7.2–7.4. In contrast, the short-term exposure test used 5% DMSO/saline solution as the delivery vehicle, without any culture medium. There was no buffering action, and the introduction of latanoprost acid could have led to a local reduction in pH. pRCECs, which are non-immortalized cells obtained from primary cultures of rabbit corneal tissue, are considered a good model for reproducing in vivo cellular reactions to irritants [28,29]. The cytotoxic effect of latanoprost acid that we detected as significant in the pRCEC test and not in the HCE-T assay can be explained by the variations in the experimental exposure parameters and the biological differences between the two cellular models. It should be noted that pH was not measured in either assay system; we hypothesize that the introduction of latanoprost acid may have led to a local reduction in pH at the cell surface, potentially contributing to the cytotoxicity detected in the pRCEC assay, and this remains to be tested directly in future studies.

Virtual docking analysis provides a mechanistic interpretation of the in vitro results by linking the qualitative toxicity predictions to a plausible molecular basis for the observed cytotoxicity. It recasts the binary in silico classifications as a coherent, structure-based account of why corneal epithelial cells lose viability in a concentration-dependent manner. This study has two limitations. First, although redocking into complex I was successful, the docking scores did not separate the free acid from the esters; the ester–acid contrast was instead captured by the ProTox-3.0 MIE call [31] and most likely reflects the poor membrane permeation of prostaglandin free acids, which is a long-recognized consideration behind the design of latanoprost as an isopropyl ester prodrug [54], rather than weaker target engagement. Second, because the scores failed to reproduce the experimental potency ranking, the SIRT3 findings were interpreted only at the binding region level. Despite these caveats, the docking analysis offers a falsifiable mechanistic framework: it clarifies how these analogs may acquire cytotoxicity [34,35,36,37] and charts the experiments required for target-level validation [33].

### 3.5. External Validation and Statistical Power Considerations

The external validation cohort of 70 molecules (52 active and 18 inactive) provided another way to assess the capability of the model to predict data that were completely different from the data used for training. It should be clear that the local model has acceptable performance on new data, but it should not be considered a definitive tool for model comparison. Statistically, the composition of the external test set, with a large difference between the number of positive (*n* = 52) and negative (*n* = 18) compounds, presented a specific problem. In classification tasks, the overall accuracy is strongly influenced by predicting the majority class. Thus, a model might have high accuracy only by predicting positive instances, even if it is weak in the minority negative class. A specificity of 0.778, derived from only 18 negative samples, has a 95% confidence interval (calculated using exact binomial methods) of approximately 0.52–0.94. This interval does not allow for a firm conclusion regarding the false-positive rate of the model. This statistical fact also supports our opinion that the external test set works best as an opening test and not as a solid basis for good validation. The same caution applies to the cross-validated specificity estimates (e.g., 0.9714 (95% CI: 0.9068–1.0000) for RF + RDKit). Because the negative class is small in every fold, these estimates carry substantial uncertainty and indicate consistency across folds rather than precise generalization performance.

### 3.6. Novelty and Contribution of the Present Study

We would like to carefully assess our novelty claims to avoid overestimating our contribution. Local QSAR modeling has been used for many toxicological endpoints for more than 20 years [55]. Its later use in areas such as skin sensitization [56,57], hepatotoxicity [58], and mutagenicity [59] has set a methodological precedent. The theoretical advantages of local modeling have been documented in cheminformatics. Among them, we have a higher predictive accuracy for the specific region of the structure, greater interpretability achieved by the analysis of the structural analogs, and the capacity to use the principles of chemical similarity [60].

To the best of our knowledge, this study is the first to systematically use scaffold-focused localized modeling to predict the ocular toxicity of PGF2α analogs, which was supported by in vitro experiments. The novelty of this study is not in the localized modeling technique (a well-known technique) but rather in the particular fusion of computational and experimental strategies for a class of compounds to solve the practically relevant problem of pharmaceutical quality control. The impurity threshold for 15(S)-latanoprost (0.5%) was set based on the usual toxicological evaluations, which could have missed the complete ocular surface toxicity profile of this stereoisomer [61,62]. This study demonstrates how localized modeling can help improve the current safety evaluation protocols for pharmaceutical impurities. We show that a localized model correctly forecasts the toxicity of this impurity, which is a task where general-purpose models fail, and provide independent experimental evidence to support this conclusion.

### 3.7. Implications for Pharmaceutical Quality Control and Regulatory Considerations

The results of this study provide useful insights for controlling the quality of ophthalmic solutions containing prostaglandin analogs, particularly regarding impurities arising from drug manufacturing. Indeed, the existing pharmacopoeial rules, for example, those of the USP-NF and the European Pharmacopoeia, mention the acceptable levels for the main latanoprost process impurities: not more than 0.5% for 15(S)-latanoprost and not more than 0.3% for *trans*-5,6-latanoprost. These limits were set by traditional toxicological evaluations, which may not have been elaborated to completely evaluate the possible toxicity of these stereoisomers on the ocular surface. This study provides additional data showing that the above impurity limits should be re-evaluated, as both the compounds shown here have in vitro cytotoxic effects.

The clinical significance of the cytotoxicity observed in vitro should be carefully evaluated. Data from pharmacokinetic studies indicate that drug levels in the corneal epithelium after topical application generally reach concentrations of 0.01–0.02%. Concentrations in the tear film drop quickly within minutes because of lacrimal drainage and dilution. In the normal case of the dosing protocols, the real concentration of the impurities that would touch the corneal epithelium would remain well below the IC50, which was determined in this study (86.22 µM for 15(S)-latanoprost and 106.70 µM for *trans*-5,6-latanoprost). However, in a person with reduced tear turnover, a weakened corneal barrier, or more frequent dosing schedules, the local concentration could be increased by a factor of 2 to 5. This could bring them closer to cytotoxic limits. The modest (approximately 1.3-fold) difference between the IC50 values of 15(S)-latanoprost and latanoprost in the HCE-T assay, although not subjected to formal statistical testing and therefore interpreted as a trend, is directionally consistent with the higher toxicological concern assigned to this impurity by the local models.

In view of the present evidence, we do not suggest changes to the existing limits; these determinations require comprehensive toxicological studies, including long-term animal studies and human clinical safety data [63,64]. The predictions that can be obtained from the localized model, as well as the supporting in vitro experimental results, suggest a possible issue that should be carefully investigated but not a demonstration that one exceeds the regulation. The process of setting the regulation thresholds should consider many factors, different health endpoints, the length of period of exposure, specificities of the patient group, and, of course, overall benefit–risk analyses, which are not fully considered by the computational simulations and short in vitro tests [65].

This work thus shows once more how local scaffold-based toxicity prediction models could be used in the workflows for the assessment of pharmaceutical impurities. Although the ICH M7 framework that is in force at the moment puts in the foreground the use of (quantitative) structure–activity relationship ((Q)SAR) methodologies for the preliminary screening of mutagenic impurities [66], standardized approaches for the assessment of general toxicological risks from process-related impurities are not so well defined. Our results show that scaffold-focused local models built with carefully assembled data on molecules with a similar degree of structurality could be a very useful supplementary method for identifying potentially dangerous impurities that could be lost by more general models. It is important to note that the intended role is “supplementary”: these local models are proposed to improve and not to replace the current safety evaluation procedure, particularly for chemical families that are not well represented in the training data of the global models.

## 4. Materials and Methods

### 4.1. Chemicals

Latanoprost (CAS 130209-82-4, chemical formula C_26_H_40_O_5_) was purchased from the Greenpine Pharmaceutical Group Co., Ltd. (Tianjin, China). The reference impurities, latanoprost acid, 15(S)-latanoprost, and *trans*-5,6-latanoprost, were purchased from CATO Research Chemicals Inc. (Guangzhou, China). The purity of all compounds was determined to be ≥95% using high-performance liquid chromatography (HPLC) analysis. All compounds were stored at −20 °C in the dark until use.

### 4.2. Construction of the Prediction Models

#### 4.2.1. Data Collection and Preprocessing

Ocular toxic and non-toxic compounds were systematically collected from the published literature and public databases [19,67,68,69,70], with dataset annotation performed according to the labeling criteria defined in the original sources. Given the limited granularity of ocular toxicity sub-classifications in publicly available datasets, both ocular irritants and corrosives were uniformly categorized as positive samples. Accordingly, a binary classification model was developed to enable early-stage screening of potential ocular toxicity risks.

To ensure data quality and structural consistency, the following preprocessing steps were applied: (1) molecular structures were standardized using RDKit (v2023.03.1) [71,72], including normalization of tautomeric forms, neutralization of charges, and removal of stereochemistry where unspecified; (2) compounds with invalid, corrupted, or missing SMILES strings were removed; (3) duplicate structures and molecules with conflicting labels were eliminated (conflicts were resolved by exclusion); (4) compounds with molecular weights below 30 Da or above 2000 Da were excluded to filter out fragments and macromolecules; and (5) elemental substances, mixtures, and inorganic compounds lacking carbon frameworks were removed. Following these procedures, a curated dataset comprising 6187 compounds was obtained, comprising 4798 positive (ocular toxicity) and 1389 negative (non-toxic) samples.

To construct a structurally focused subset relevant to prostaglandin analogs and related scaffold classes, the complete dataset was screened using two parallel filtering strategies: (i) scaffold-based filtering, in which the Murcko scaffold of PGF2α was extracted using RDKit’s MurckoScaffoldSmiles function [73], and molecules sharing structurally similar Murcko scaffolds with PGF2α were retrieved, and (ii) fingerprint similarity screening, whereby Tanimoto similarity was calculated between each molecule and PGF2α based on 166-bit MACCS fingerprints [74], and a similarity threshold of ≥0.5 was applied. The results from both filtering strategies were merged and deduplicated, yielding a locally focused dataset of 350 compounds (262 positive and 88 negative) enriched with prostaglandin-like scaffolds for targeted model evaluation (Table S1).

#### 4.2.2. Molecular Feature Representation

SMILES strings were converted into molecular graph objects using the RDKit (v2023.03.1) [71]. Two categories of molecular representations were computed for model development: 2D molecular fingerprints and graph representations for deep learning. Three types of binary fingerprints were generated: (i) RDKit fingerprints, which are hashed topological fingerprints with a default path length of up to seven bonds and a fingerprint size of 2048 bits; (ii) MACCS keys, which are 166 predefined structural key descriptors capturing common chemical substructures [74]; and (iii) Morgan (circular) fingerprints, which are extended-connectivity fingerprints with a radius of two bonds and a fingerprint size of 2048 bits, equivalent to the ECFP4 fingerprints. For the GCN, each molecule is represented as an undirected graph G = (V, E), where V denotes the set of atoms (nodes) and E denotes the set of bonds (edges). Node features were initialized using a concatenated vector comprising one-hot encoded atom type (11 classes: C, N, O, S, F, Cl, Br, I, P, B, and other), atom degree (one-hot encoded, 6 classes), formal charge, number of radical electrons, hybridization state (one-hot encoded: sp, sp^2^, sp^3^, sp^3^d, and sp^3^d^2^), aromaticity (binary), and the number of attached hydrogens (one-hot encoded, 5 classes), yielding a 37-dimensional initial node feature vector for each atom. Edge features encoded bond type (single, double, triple, aromatic).

#### 4.2.3. Machine Learning Methods

All models were developed using Python (v3.10). Conventional machine learning models were implemented using scikit-learn (v1.3.0) [75] and XGBoost (v2.0.0), including RF, an ensemble of decision trees trained via bootstrap aggregating with Gini impurity as the splitting criterion; XGBoost, gradient-boosted decision trees optimized through regularized objective minimization; SVM, with the kernel (rbf, linear, or poly) and regularization parameter C selected by grid search (Table 7); and KNN, instance-based classification using feature-space distance metrics.

The CNN model was implemented using PyTorch (v2.1.0). The architecture consists of two sequential convolutional blocks: The first block contained a 1D convolutional layer (kernel_size optimized by grid search over {8, 16, 32}, output channels = 64), ReLU activation, batch normalization, and max pooling (pool_size = 4). The second block contained a 1D convolutional layer (kernel_size = 8, output channels = 128), ReLU activation, batch normalization, and max pooling (pool_size = 2). The output of the convolutional blocks was flattened through a Flatten layer and then fed into one fully connected hidden layer with 128 units, using ReLU activation and dropout regularization (dropout rate = 0.3) followed by batch normalization before connecting to the final output layer. The output layer consists of one neuron with sigmoid activation for binary classification. The model uses the Adam optimizer with a learning rate optimized via grid search over {0.001, 0.0005, 0.0001}, and the loss function is a binary cross-entropy.

The GCN was implemented using PyTorch Geometric (v2.4.0). The architecture employed standard GCNConv layers from PyTorch Geometric for message passing. The number of convolutional layers (2–3) and the hidden dimension (64, 128, or 256, uniform across layers) were optimized via grid search under 10-fold stratified cross-validation; each convolutional layer was followed by batch normalization, rectified linear unit activation, and a dropout. Node embeddings from the final convolutional layer were aggregated via global mean pooling to yield a graph-level representation, which was then passed through two fully connected layers—a hidden layer (dimension = hidden_dim/2) with ReLU activation and dropout followed by an output layer for binary classification. The dropout rate (0.2–0.4) was optimized using a grid search. Models were trained with the Adam optimizer (weight decay = 1 × 10^−4^; learning rate selected from {0.01, 0.005, 0.001}) coupled with a ReduceLROnPlateau scheduler (patience = 8, factor = 0.5, minimum learning rate = 1 × 10^−5^) using binary cross-entropy loss (batch size = 32) for up to 100 epochs with early stopping (patience = 15 epochs based on the validation AUC).

#### 4.2.4. Dataset Partitioning and Cross-Validation

The curated dataset was partitioned into a training set and an external hold-out test set at a ratio of 8:2 using stratified random sampling to preserve the positive-to-negative class ratio in both subsets. Data splitting was performed using a fixed random seed (random_state = 42) to ensure reproducibility. The training set was used for model fitting and hyperparameter optimization, whereas the external test set was reserved exclusively for the final model evaluation and was never accessed during training or validation.

For hyperparameter tuning, 10-fold stratified cross-validation was applied to the training dataset. In each fold, the training data were further split into nine training folds and one validation fold, maintaining class distribution consistency across all folds.

#### 4.2.5. Hyperparameter Optimization

Hyperparameter optimization was performed using a grid search with 10-fold cross-validation via GridSearchCV in scikit-learn. The hyperparameter search spaces for each algorithm are presented in Table 7. The optimization was performed in two stages: an initial coarse grid search followed by a refinement search around the best-performing configurations. The ranges listed in Table 7 represent the complete search space covered across both stages.

For deep learning models, hyperparameters including the learning rate, batch size, number of layers, hidden dimensions, and dropout rate were tuned using manual grid search. The optimal hyperparameters were determined by maximizing the average cross-validation AUC.

#### 4.2.6. Model Evaluation Metrics

The following evaluation metrics were employed to comprehensively assess the model performance:

Accuracy (ACC)—the proportion of correctly classified instances among all instances:

$$ACC=\frac{TP+TN}{TP+TN+FP+FN}\;$$

Sensitivity (SEN)—the true-positive rate, which measures the proportion of actual positives correctly identified:

$$SEN=\frac{TP}{TP+FN}\;$$

Specificity (SP)—the true-negative rate, which measures the proportion of actual negatives correctly identified:

$$SP=\frac{TN}{TN+FP}\;$$

Balanced accuracy (BA)—the arithmetic mean of sensitivity and specificity, accounting for class imbalance:

$$BA=\frac{SEN+SP}{2}\;$$

Precision (P)—the proportion of positive predictions that are true positives:

$$P=\frac{TP}{TP+FP}\;$$

F1 Score (F1)—the harmonic mean of precision and sensitivity:

$$F1=\frac{2\times P\times SEN}{P+SEN}\;$$

Matthews correlation coefficient (MCC)—a balanced measure of classification quality that accounts for all four categories of the confusion matrix:

$$MCC=\frac{TP\times TN-FP\times FN}{\sqrt{\left(TP+FP\right)\left(TP+FN\right)\left(TN+FP\right)\left(TN+FN\right)}}\;$$

#### 4.2.7. Applicability Domain Assessment Methods

For the fingerprint-based models (RF, XGB, SVM, KNN and CNN), a leverage-based method was employed according to OECD guidance [76]. The leverage (h) of a query compound was calculated as follows:

$$h_{i}=x_{i}^{T}{\left(X^{T}X\right)}^{-1}x_{i}$$

where x_i_ is the standardized PCA-reduced feature vector of the query compound and X is the corresponding matrix of the training set. The warning leverage threshold was defined as h* = 3(p + 1)/n, where p is the number of retained principal components and n is the number of training samples. Compounds with hi > h* were considered to be outside the AD.

For the GCN model, a graph embedding-based K-nearest neighbor (KNN) method was applied. The trained GCN encoder was used to generate graph-level embedding vectors for both the training and query compounds. The Euclidean distance from each query compound to its 5-nearest neighbors (5-NN) in the training set was computed in the embedding space. The AD threshold was set at the 95th percentile of the 5-NN distances in the training set. Query compounds exceeding this threshold were flagged as being outside the AD.

#### 4.2.8. Y-Randomization (Y-Scrambling) Validation

Y-randomization was performed to assess the statistical robustness of the developed models and rule out chance correlation, in accordance with OECD QSAR Validation Principle 4 (a measure of goodness-of-fit, robustness, and predictivity). The response labels (ocular toxic/non-toxic) of the training set were randomly permuted 100 times, preserving the original class distribution. For each randomized instance, the optimal model was retrained using the same hyperparameters and feature representations as the original models. The mean AUC value across all 100 randomized runs was computed and compared with the AUC of the original (non-randomized) model. A model was considered robust if the mean randomized AUC was substantially lower than the original AUC (typically by >0.2) and the original AUC exceeded the 95th percentile of the randomized AUC distribution. Y-randomization was performed for the best-performing RF and XGBoost models on both the full dataset and prostaglandin-focused subset.

#### 4.2.9. GCN Gradient Saliency Analysis

To interpret the predictions of the GCN model at the atomic level, a gradient-based saliency analysis was performed to identify the atoms and substructures that contributed the most to the ocular toxicity prediction. The node feature matrix was defined as a differentiable tensor and fed into the trained GCN. The gradient of the predicted toxicity probability with respect to each node’s input features was computed using backpropagation. The L2 norm of the gradient vector for each atom was computed as the atom-importance score:

$$S_{i}={∥\frac{\partial \hat{y}}{\partial x_{i}}∥}_{2}$$

where S_i_ is the saliency score for atom i, ŷ is the predicted toxicity probability, and x_i_ is the input feature vector of atom i. Higher saliency scores indicate that the atoms exert a greater influence on the model’s prediction. The atom-level saliency scores were mapped onto 2D molecular structures for visual interpretation, highlighting substructural motifs potentially associated with ocular toxicity.

### 4.3. Cell Culture

#### 4.3.1. HCE-T Cell Culture

Human corneal epithelial cells (HCE-T, ATCC CRL-11515) were purchased from Procell Life Science & Technology Co., Ltd. (Wuhan, China). Cells were maintained in high-glucose Dulbecco’s Modified Eagle Medium (DMEM, Gibco, Grand Island, NY, USA) supplemented with 15% (*v*/*v*) fetal bovine serum (FBS, Gibco), 1% (*v*/*v*) penicillin–streptomycin (P/S, 100 U/mL penicillin and 100 μg/mL streptomycin, Gibco), 5 μg/mL recombinant human insulin (Sigma-Aldrich, Saint Louis, MO, USA), and 10 ng/mL human epidermal growth factor (hEGF, PeproTech, Cranbury, NJ, USA). Cells were cultured at 37 °C in a humidified atmosphere containing 5% CO_2_. The medium was renewed every 2–3 days. Cells at passage 5 were used in the experiments to ensure phenotypic consistency.

#### 4.3.2. pRCEC Culture

Primary rabbit corneal epithelial cells (pRCECs) were purchased from the Meisen Chinese Collection of Cell Cultures (Hangzhou, China). Cells were cultured in epithelial cell medium (EpiCM, ScienCell Research Laboratories, Carlsbad, CA, USA) supplemented with 10% fetal bovine serum (Meisen, Shenzhen, China), 1% penicillin/streptomycin (Meisen), and 1% epithelial cell growth supplement (Meisen) and maintained at 37 °C in a humidified atmosphere with 5% CO_2_. Cells at passage 3 were used for cytotoxicity assays.

### 4.4. Corneal Cytotoxicity Assays

#### 4.4.1. CCK-8 Assay

pRCECs were seeded into 96-well plates at a density of 3 × 10^3^ cells per well in 100 μL of complete EpiCM and cultured for 5 days to achieve a confluent epithelial monolayer. Prior to compound exposure, the culture medium was aspirated and replaced with a serum-free medium. Latanoprost and its impurity standards were dissolved in DMSO to prepare stock solutions, which were subsequently diluted to working concentrations of 0.05% and 0.005% (*w*/*v*) in saline; the final vehicle (DMSO) concentration in all working solutions was 5% (*v*/*v*). Cells were exposed to the test compounds for 5 min at 37 °C to simulate a single topical ocular administration event following the short time exposure (STE) principle [26,27]. The experimental setup included the following control groups: complete culture medium as the blank control; equal volume of cell suspension as the culture medium control; 5% (*v*/*v*) DMSO in normal saline as the solvent control; and 0.01% (*w*/*v*) sodium lauryl sulfate (SLS) in normal saline as the positive control. Following exposure, the compound-containing medium was removed, the cells were washed twice with phosphate-buffered saline (PBS; pH 7.4), and fresh complete medium was added. Cell viability was assessed using the Cell Counting Kit-8 (CCK-8; Dojindo Laboratories, Rockville, MD, USA). CCK-8 reagent (10 μL per 100 μL medium) was added to each well, and the plates were incubated at 37 °C for 4 h in the dark. Absorbance at 450 nm was measured using a microplate reader (SpectraMax iD3, Molecular Devices, San Jose, CA, USA). Cell viability was calculated as follows:

$$\mathrm{Cell}\;\mathrm{viability}\;(\%)=\frac{A_{\mathrm{sample}}-A_{\mathrm{blank}}}{A_{\mathrm{control}}-A_{\mathrm{blank}}}\times 100\%$$

where A_sample_, A_control_, and A_blank_ represent the absorbance of the test compound, vehicle control, and medium-only blank wells, respectively. Each treatment was performed in at least six replicate wells, and the experiments were independently repeated three times.

#### 4.4.2. MTT Assay

HCE-T cells were seeded into 96-well plates at a density of 6 × 10^3^ cells per well in 100 μL of complete DMEM and incubated overnight at 37 °C with 5% CO_2_ to allow cell adhesion. Latanoprost and its impurity standards were dissolved in DMSO and diluted to the desired concentrations. Latanoprost, 15(S)-latanoprost, and *trans*-5,6-latanoprost were tested at 50, 75, 100, 125, 150, 175, 200, and 225 µM, whereas latanoprost acid was tested at 0.2, 0.4, 0.6, 0.8, and 1.0 mM concentrations. Cells were treated with the test compounds for 24 h at 37 °C, with 0.5% DMSO in the culture medium as the vehicle control. Following incubation, the medium was removed, and MTT reagent (5 mg/mL in PBS, 20 μL per well; Sigma-Aldrich) was added to each well. The plates were incubated at 37 °C for 4 h in the dark. The resulting formazan crystals were dissolved in 150 μL of DMSO per well, and the plates were agitated on an orbital shaker for 10 min at room temperature. Absorbance at 570 nm was measured using a microplate reader (SpectraMax iD3, Molecular Devices). Cell viability was calculated relative to that of the vehicle control, as described for the CCK-8 assay, using the following formula:

$$\mathrm{Cell}\;\mathrm{viability}\;(\%)=\frac{A_{\mathrm{sample}}-A_{\mathrm{blank}}}{A_{\mathrm{control}}-A_{\mathrm{blank}}}\times 100\%$$

Each treatment was performed in three replicate wells, and experiments were independently repeated three times.

#### 4.4.3. Statistical Analysis

All quantitative data are presented as the mean ± standard deviation (SD) of at least three independent experiments. Statistical comparisons between multiple groups were performed using one-way analysis of variance (ANOVA) followed by Tukey’s honestly significant difference (HSD) post hoc test for pairwise comparisons. Statistical significance was set at *p <* 0.05. All statistical analyses were performed using GraphPad Prism v8.5. Dose–response relationships were analyzed using nonlinear regression to determine the IC_50_.

### 4.5. Virtual Molecular Docking

Putative protein targets were identified by ligand-based prediction using SwissTargetPrediction (Homo sapiens; the top 15 targets per compound, probability ≥0.01) [32] and ProTox-3.0 profiling of toxicity endpoints and molecular initiating events (MIEs) [31]. Cross-referencing these predictions with published mechanistic studies identified mitochondrial complex I and human SIRT3 as key targets [34,35,36,37]. Molecular docking of latanoprost and its impurities to complex I (PDB: 6ZKC [37,77,78]; supernumerary subunits and co-crystallized decylubiquinone removed) and SIRT3 (PDB: 5H4D; NAD^+^ retained, amiodarone removed) was performed using AutoDock Vina 1.2.7 [79,80,81]. Prior to docking, the protein structures were prepared in AutoDockTools by adding polar hydrogen atoms and assigning charges, and ligand three-dimensional conformers bearing the correct stereochemistry were energy-minimized. The grid boxes were centered on the coordinates of the crystallographic ligands, encompassing the quinone-binding channel of complex I and the catalytic cleft of SIRT3, respectively. The reliability of the docking protocol was validated by redocking the co-crystallized ligands and calculating the heavy-atom RMSD values relative to their experimental poses. Binding interactions (hydrogen bonds, donor–acceptor distance <3.6 Å; hydrophobic contacts, carbon–carbon distance <4.5 Å) were analyzed and visualized using PyMOL 3.1 (Schrödinger LLC, New York, NY, USA) [82].

## 5. Conclusions

This study shows that scaffold-specific local structure–toxicity relationship (STR) models, trained with a carefully selected dataset of 350 structurally similar PGF2α analogs, provide reliable predictions of ocular toxicity in their applicability domain. Global modeling was not applicable to any of the four compounds. The refined RF model with RDKit molecular descriptors yielded a balanced accuracy of 0.9405 ± 0.0614 for 10-fold cross-validation and an AUC of 0.9861 on the external test set. The model was also shown to be robust by Y-randomization analysis, with a Z-score of >3.5 (*p* < 0.001). Experimental validation using in vitro assays, short-term exposure tests on primary rabbit corneal epithelial cells (pRCECs), and continuous exposure assays on HCE-T cells revealed a clear concentration-dependent effect. None of the four compounds showed toxicity at a therapeutic concentration of 0.005%. A significant increase in cytotoxicity was observed at 0.05% (*p* < 0.001). Among the analyzed impurity compounds, 15(S)-latanoprost exhibited the highest cytotoxic potential, with a half-maximal inhibitory concentration (IC_50_) of 86.22 µM. This value was approximately 1.3 times lower (more potent) than the IC_50_ of latanoprost (112.60 µM). We did not formally test this difference and read it as a trend; however, even as a trend it flags a stereoisomeric impurity that quality specifications should not overlook. Three independent computational lines, ligand-based target prediction, ProTox-3.0 MIE profiling, and molecular docking, pointed to the same organelle. All four analogs fit the quinone-binding channel of complex I (−9.51 to −10.55 kcal/mol) and the catalytic-cleft region of SIRT3 (−6.54 to −7.25 kcal/mol). Whether C15 epimerization alters mitochondrial engagement directly cannot be resolved by the present docking scores and requires target-level experimental validation.

Regarding stereoisomeric impurities, the failure of the two-dimensional GCN architectures to discriminate between stereoisomers (both get the same predicted probability of 0.9956) is a methodological observation that shows a limit of the graph-based representations of chiral pharmaceutical molecules. Rather than being a mere limitation, this shows exactly where chirality-aware descriptors and three-dimensional models have the most to offer for chiral drug molecules. Overall, these results indicate that local modeling, together with focused in vitro validation, is a practical supplementary approach within the IATA framework for evaluating the impurity risks of certain classes of compounds [83,84]. Wider external validation, GHS-resolved models, and prospective testing would bring this strategy closer to routine regulatory use.

## Figures and Tables

**Table 1:** Best hyperparameter combinations for the local models (*n* = 350).

Models		Best Hyperparameter Combinations
No	Name	
1	KNN + RDKit	metric: cosine, n_neighbors: 9, weights: distance
2	KNN + MACCS	metric: cosine, n_neighbors: 31, weights: distance
3	KNN + Morgan	metric: cosine, n_neighbors: 31, weights: distance
4	SVM + RDKit	C: 10, gamma: scale, kernel: rbf
5	SVM + MACCS	C: 0.01, gamma: scale, kernel: linear
6	SVM + Morgan	C: 0.01, gamma: scale, kernel: linear
7	RF + RDKit	max_depth: 10, max_features: sqrt, min_samples_leaf: 2, min_samples_split: 3, n_estimators: 300
8	RF + MACCS	max_depth: 5, max_features: sqrt, min_samples_leaf: 1, min_samples_split: 7, n_estimators: 300
9	RF + Morgan	max_depth: None, max_features: log2, min_samples_leaf: 1, min_samples_split: 5, n_estimators: 500
10	XGBoost + RDKit	learning_rate: 0.1, max_depth: 6, n_estimators: 50, subsample: 0.6
11	XGBoost + MACCS	learning_rate: 0.1, max_depth: 2, n_estimators: 50, subsample: 0.6
12	XGBoost + Morgan	learning_rate: 0.05, max_depth: 6, n_estimators: 400, subsample: 1.0
13	CNN + RDKit	kernel_size: 16, lr: 0.0005
14	CNN + MACCS	kernel_size: 32, lr: 0.001
15	CNN + Morgan	kernel_size: 16, lr: 0.001
16	GCN	hidden_dim: 64, dropout: 0.2, lr: 0.005, num_layers: 3

**Table 2:** Performance comparison of the local models (*n* = 350) under 10-fold cross-validation.

Models		Metrics							
No	Name	AUC (95% CI)	ACC (95% CI)	SEN (95% CI)	SP (95% CI)	P (95% CI)	F1 (95% CI)	BA (95% CI)	MCC (95% CI)
1	KNN + RDKit	0.9225 (0.8783, 0.9666)	0.9214 (0.8837, 0.9592)	0.9286 (0.8701, 0.9870)	0.9000 (0.8031, 0.9969)	0.9688 (0.9387, 0.9988)	0.9452 (0.9167, 0.9738)	0.9143 (0.8724, 0.9562)	0.8177 (0.7431, 0.8923)
2	KNN + MACCS	0.9323 (0.8789, 0.9857)	0.9464 (0.9163, 0.9766)	0.9572 (0.9197, 0.9946)	0.9143 (0.8281, 1.0000)	0.9728 (0.9461, 0.9995)	0.9637 (0.9428, 0.9846)	0.9357 (0.8939, 0.9775)	0.8686 (0.7964, 0.9407)
3	KNN + Morgan	0.8973 (0.8514, 0.9432)	0.8393 (0.8007, 0.8778)	0.8238 (0.7681, 0.8795)	0.8857 (0.7918, 0.9796)	0.9589 (0.9279, 0.9900)	0.8835 (0.8527, 0.9142)	0.8548 (0.8112, 0.8983)	0.6516 (0.5740, 0.7291)
4	SVM + RDKit	0.9517 (0.9154, 0.9880)	0.9250 (0.8792, 0.9708)	0.9238 (0.8633, 0.9843)	0.9286 (0.8417, 1.0000)	0.9770 (0.9495, 1.0000)	0.9471 (0.9133, 0.9809)	0.9262 (0.8791, 0.9733)	0.8296 (0.7336, 0.9257)
5	SVM + MACCS	0.9497 (0.9154, 0.9839)	0.9429 (0.9106, 0.9752)	0.9572 (0.9197, 0.9946)	0.9000 (0.8310, 0.9690)	0.9672 (0.9452, 0.9891)	0.9613 (0.9388, 0.9837)	0.9286 (0.8901, 0.9671)	0.8570 (0.7789, 0.9352)
6	SVM + Morgan	0.9469 (0.9132, 0.9807)	0.9357 (0.9123, 0.9592)	0.9381 (0.9058, 0.9704)	0.9285 (0.8747, 0.9824)	0.9761 (0.9581, 0.9942)	0.9559 (0.9393, 0.9725)	0.9334 (0.9070, 0.9597)	0.8431 (0.7880, 0.8983)
7	RF + RDKit	0.9612 (0.9282, 0.9942)	0.9250 (0.8679, 0.9821)	0.9095 (0.8334, 0.9856)	0.9714 (0.9068, 1.0000)	0.9909 (0.9703, 1.0000)	0.9450 (0.9016, 0.9884)	0.9405 (0.8942, 0.9868)	0.8453 (0.7352, 0.9553)
8	RF + MACCS	0.9401 (0.8887, 0.9916)	0.9500 (0.9226, 0.9775)	0.9619 (0.9351, 0.9888)	0.9143 (0.8281, 1.0000)	0.9727 (0.9455, 0.9998)	0.9665 (0.9482, 0.9848)	0.9381 (0.8947, 0.9815)	0.8724 (0.7998, 0.9449)
9	RF + Morgan	0.9633 (0.9261, 1.0000)	0.9429 (0.9084, 0.9774)	0.9476 (0.9038, 0.9915)	0.9285 (0.8747, 0.9824)	0.9761 (0.9580, 0.9942)	0.9606 (0.9362, 0.9849)	0.9381 (0.9048, 0.9714)	0.8633 (0.7836, 0.9429)
10	XGBoost + RDKit	0.9605 (0.9237, 0.9974)	0.9464 (0.9118, 0.9810)	0.9619 (0.9267, 0.9971)	0.9000 (0.8159, 0.9841)	0.9677 (0.9407, 0.9947)	0.9639 (0.9404, 0.9875)	0.9310 (0.8845, 0.9774)	0.8638 (0.7756, 0.9519)
11	XGBoost + MACCS	0.9449 (0.9018, 0.9880)	0.9465 (0.9189, 0.9740)	0.9667 (0.9386, 0.9947)	0.8857 (0.8051, 0.9663)	0.9633 (0.9380, 0.9887)	0.9643 (0.9458, 0.9828)	0.9262 (0.8849, 0.9675)	0.8612 (0.7885, 0.9339)
12	XGBoost + Morgan	0.9599 (0.9228, 0.9970)	0.9500 (0.9177, 0.9823)	0.9667 (0.9437, 0.9897)	0.9000 (0.7917, 1.0000)	0.9688 (0.9353, 1.0000)	0.9669 (0.9462, 0.9877)	0.9333 (0.8778, 0.9889)	0.8682 (0.7766, 0.9598)
13	CNN + RDKit	0.9259 (0.8784, 0.9733)	0.8964 (0.8522, 0.9406)	0.8905 (0.8371, 0.9439)	0.9143 (0.8156, 1.0000)	0.9714 (0.9384, 1.0000)	0.9270 (0.8957, 0.9583)	0.9024 (0.8495, 0.9553)	0.7637 (0.6584, 0.8690)
14	CNN + MACCS	0.9048 (0.8532, 0.9563)	0.9143 (0.8868, 0.9418)	0.9524 (0.9246, 0.9802)	0.8000 (0.6901, 0.9099)	0.9373 (0.9032, 0.9715)	0.9436 (0.9259, 0.9612)	0.8762 (0.8249, 0.9275)	0.7729 (0.6923, 0.8534)
15	CNN + Morgan	0.9483 (0.9140, 0.9827)	0.9072 (0.8727, 0.9417)	0.8905 (0.8347, 0.9462)	0.9571 (0.9078, 1.0000)	0.9859 (0.9696, 1.0000)	0.9335 (0.9070, 0.9600)	0.9238 (0.9029, 0.9448)	0.7999 (0.7380, 0.8619)
16	GCN	0.9293 (0.8955, 0.9630)	0.9143 (0.8868, 0.9418)	0.9334 (0.9004, 0.9663)	0.8571 (0.7890, 0.9252)	0.9525 (0.9306, 0.9743)	0.9420 (0.9230, 0.9610)	0.8953 (0.8601, 0.9304)	0.7825 (0.7135, 0.8515)

**Table 3:** Performance comparison of the local models (*n* = 350) under external test set.

Models		Metrics							
No	Name	AUC (95% CI)	ACC (95% CI)	SEN (95% CI)	SP (95% CI)	P (95% CI)	F1 (95% CI)	BA (95% CI)	MCC (95% CI)
1	KNN + RDKit	0.9722 (0.9295, 1.0000)	0.9429 (0.8571, 1.0000)	0.9615 (0.8333, 1.0000)	0.8889 (0.8000, 1.0000)	0.9615 (0.9273, 1.0000)	0.9615 (0.8973, 1.0000)	0.9252 (0.8705, 1.0000)	0.8504 (0.6785, 1.0000)
2	KNN + MACCS	0.9765 (0.9407, 0.9965)	0.8857 (0.8286, 0.9857)	0.8462 (0.7754, 1.0000)	1.0000 (0.8570, 1.0000)	1.0000 (0.9423, 1.0000)	0.9167 (0.8706, 0.9905)	0.9231 (0.8796, 0.9894)	0.7654 (0.6605, 0.9611)
3	KNN + Morgan	0.9466 (0.8845, 0.9904)	0.8286 (0.7714, 0.9571)	0.7885 (0.7115, 1.0000)	0.9444 (0.6954, 1.0000)	0.9762 (0.8888, 1.0000)	0.8723 (0.8260, 0.9739)	0.8665 (0.8003, 0.9530)	0.6539 (0.5294, 0.8940)
4	SVM + RDKit	0.9861 (0.9588, 1.0000)	0.9571 (0.9000, 1.0000)	0.9423 (0.8749, 1.0000)	1.0000 (1.0000, 1.0000)	1.0000 (1.0000, 1.0000)	0.9703 (0.9333, 1.0000)	0.9712 (0.9363, 1.0000)	0.8987 (0.7781, 1.0000)
5	SVM + MACCS	0.9519 (0.8969, 0.9943)	0.9286 (0.8429, 0.9857)	0.9615 (0.8392, 1.0000)	0.8333 (0.6875, 1.0000)	0.9434 (0.8888, 1.0000)	0.9524 (0.8888, 0.9905)	0.8974 (0.8208, 0.9783)	0.8102 (0.6279, 0.9594)
6	SVM + Morgan	0.9722 (0.9109, 1.0000)	0.9714 (0.9286, 1.0000)	0.9808 (0.9423, 1.0000)	0.9444 (0.8095, 1.0000)	0.9808 (0.9361, 1.0000)	0.9808 (0.9524, 1.0000)	0.9626 (0.8965, 1.0000)	0.9252 (0.8169, 1.0000)
7	RF + RDKit	0.9861 (0.9611, 1.0000)	0.9429 (0.8714, 1.0000)	0.9423 (0.8298, 1.0000)	0.9444 (0.8571, 1.0000)	0.9800 (0.9434, 1.0000)	0.9608 (0.9070, 1.0000)	0.9434 (0.9048, 1.0000)	0.8579 (0.7229, 1.0000)
8	RF + MACCS	0.9797 (0.9451, 1.0000)	0.9143 (0.8429, 1.0000)	0.9038 (0.7872, 1.0000)	0.9444 (0.8182, 1.0000)	0.9792 (0.9318, 1.0000)	0.9400 (0.8810, 1.0000)	0.9241 (0.8854, 1.0000)	0.7986 (0.6719, 1.0000)
9	RF + Morgan	0.9861 (0.9599, 1.0000)	0.9571 (0.8429, 1.0000)	0.9808 (0.8036, 1.0000)	0.8889 (0.8333, 1.0000)	0.9623 (0.9387, 1.0000)	0.9714 (0.8911, 1.0000)	0.9348 (0.8929, 1.0000)	0.8864 (0.6826, 1.0000)
10	XGBoost + RDKit	0.9541 (0.8824, 0.9969)	0.9143 (0.8571, 0.9857)	0.9038 (0.8363, 1.0000)	0.9444 (0.7855, 1.0000)	0.9792 (0.9259, 1.0000)	0.9400 (0.9009, 0.9905)	0.9241 (0.8545, 0.9906)	0.7986 (0.6647, 0.9639)
11	XGBoost + MACCS	0.9573 (0.8964, 0.9973)	0.9286 (0.8429, 0.9857)	0.9423 (0.8148, 1.0000)	0.8889 (0.7500, 1.0000)	0.9608 (0.9057, 1.0000)	0.9515 (0.8846, 0.9912)	0.9156 (0.8400, 0.9906)	0.8169 (0.6436, 0.9639)
12	XGBoost + Morgan	0.9658 (0.9220, 0.9928)	0.9143 (0.8429, 0.9718)	0.9615 (0.8545, 1.0000)	0.7778 (0.6111, 1.0000)	0.9259 (0.8571, 1.0000)	0.9434 (0.8889, 0.9840)	0.8697 (0.7858, 0.9643)	0.7695 (0.5989, 0.9352)
13	CNN + RDKit	0.9659 (0.9143, 0.9976)	0.9328 (0.8714, 0.9857)	0.9313 (0.8431, 1.0000)	0.9373 (0.7997, 1.0000)	0.9774 (0.9245, 1.0000)	0.9531 (0.9024, 0.9910)	0.9343 (0.8616, 0.9906)	0.8364 (0.6719, 0.9651)
14	CNN + MACCS	0.9751 (0.9352, 1.0000)	0.9577 (0.9000, 1.0000)	0.9635 (0.8909, 1.0000)	0.9417 (0.8182, 1.0000)	0.9795 (0.9388, 1.0000)	0.9710 (0.9307, 1.0000)	0.9526 (0.8877, 1.0000)	0.8926 (0.7597, 1.0000)
15	CNN + Morgan	0.9814 (0.9503, 1.0000)	0.9266 (0.8286, 1.0000)	0.9291 (0.7777, 1.0000)	0.9203 (0.7500, 1.0000)	0.9721 (0.9166, 1.0000)	0.9482 (0.8712, 1.0000)	0.9247 (0.8540, 1.0000)	0.8285 (0.6329, 1.0000)
16	GCN	0.9082 (0.8240, 0.9754)	0.9031 (0.8425, 0.9714)	0.9638 (0.9057, 1.0000)	0.7278 (0.5000, 0.9286)	0.9110 (0.8305, 0.9815)	0.9361 (0.8868, 0.9808)	0.8458 (0.7315, 0.9485)	0.7359 (0.5447, 0.9016)

**Table 4:** AD assessment of latanoprost and its impurities using the GCN model.

Name	AD_Distance	AD_Threshold
Latanoprost	1.821	2.877
Latanoprost acid	1.899	
15(*S*)-Latanoprost	1.821	
*trans*-5,6-Latanoprost	1.821	

**Table 5:** Prediction results of latanoprost and its impurities by model (1 = toxic; 0 = non-toxic).

Models		Prediction Results			
No	Name	Latanoprost	Latanoprost Acid	15(*S*)-Latanoprost	*trans*-5,6-Latanoprost
1	KNN + RDKit	1	1	1	1
2	KNN + MACCS	1	1	1	1
3	KNN + Morgan	1	1	1	1
4	SVM + RDKit	1	1	1	1
5	SVM + MACCS	1	1	1	1
6	SVM + Morgan	0	1	0	0
7	RF + RDKit	1	1	1	1
8	RF + MACCS	1	1	1	1
9	RF + Morgan	1	1	1	1
10	XGBoost + RDKit	1	1	1	1
11	XGBoost + MACCS	1	1	1	1
12	XGBoost + Morgan	0	1	0	0
13	CNN + RDKit	1	1	1	1
14	CNN + MACCS	0	1	0	0
15	CNN + Morgan	1	1	1	1
16	GCN	1	1	1	1

Note: 1 represents toxic; 0 represents non-toxic.

**Table 6:** Analysis and statistical table for prediction results of various computational models.

Name	SMILES	Global Models ^1^	Local Models ^2^	toxCSM ^3^	
				Eye Corrosion	Eye Irritation
Latanoprost	O[C@@H](CCC1=CC=CC=C1)CC[C@H]2[C@H](O)C[C@H](O)[C@@H]2C/C=C\CCCC(OC(C)C)=O	0/16	13/16	0.0	0.0
Latanoprost acid	O[C@@H](CCC1=CC=CC=C1)CC[C@H]2[C@H](O)C[C@H](O)[C@@H]2C/C=C\CCCC(O)=O	0/16	16/16	0.0	0.0
15(*S*)-Latanoprost	O[C@H](CCC1=CC=CC=C1)CC[C@H]2[C@H](O)C[C@H](O)[C@@H]2C/C=C\CCCC(OC(C)C)=O	0/16	13/16	0.0	0.01
*trans*-5,6-Latanoprost	O[C@@H](CCC1=CC=CC=C1)CC[C@H]2[C@H](O)C[C@H](O)[C@@H]2C/C=C/CCCC(OC(C)C)=O	0/16	13/16	0.0	0.01

^1^ The general ocular toxicity prediction models were constructed based on 6187 compounds. ^2^ The specialized ocular toxicity prediction models were constructed based on 350 compounds with structural similarities to PGF_2α_. ^3^ The toxCSM platform (https://biosig.lab.uq.edu.au/toxcsm/ accessed on 10 November 2025), models constructed based on 2299 (eye corrosion) and 5220 (eye irritation) compounds). The results were expressed as the number of models yielding a positive prediction out of the total number of models built (positive/total) or toxicity confidence values between 0.0 and 1.0, where lower values indicate lower predicted toxicity 3 [41].

**Table 7:** Hyperparameter ranges for machine learning.

Model	Parameter	Search Range
RF	n_estimators	[100, 200, 300, 400, 500]
RF	max_depth	[None, 5, 10, 20, 30]
RF	min_samples_split	[3, 5, 7, 9]
RF	min_samples_leaf	[1, 2]
RF	max_features	[“sqrt”, “log2”]
XGB	n_estimators	[50, 100, 200, 400]
XGB	max_depth	[2, 4, 6, 8]
XGB	learning_rate	[0.01, 0.05, 0.1, 0.2]
XGB	subsample	[0.6, 0.8, 1.0]
SVM	C	[0.01, 0.1, 1, 10, 100]
SVM	kernel	[“rbf”, “linear”, “poly”]
SVM	gamma	[‘scale’, ‘auto’, 0.001, 0.01, 0.1]
KNN	n_neighbors	[1, 3, 5, 9, 15, 21, 31]
KNN	weights	[‘uniform’, ‘distance’]
KNN	metric	[‘euclidean’, ‘manhattan’, ‘minkowski’, ‘cosine’]
CNN	kernel_size	[8, 16, 32]
CNN	learning_rate	[0.001, 0.0005, 0.0001]
GCN	hidden_dim	[64, 128, 256]
GCN	dropout	[0.2, 0.3, 0.4]
GCN	learning_rate	[0.01, 0.005, 0.001]
GCN	num_layers	[2, 3]

## Data Availability

The original contributions presented in this study are included in the article/Supplementary Materials. Further inquiries can be directed to the corresponding author.

## Supplementary Materials

The following supporting information can be downloaded at https://www.mdpi.com/article/10.3390/molecules31173116/s1. Table S1. 350 compounds used to develop the local STR model; Table S2. SwissTargetPrediction results; Table S3. ProTox-3.0 prediction summary; Table S4. Molecular docking details; Table S5. Docking results for the four analogues and the redocked co-crystallized ligands; Figure S1. ROC curves of 16 Local STR models; unchanged from the first revision, submitted; Figure S2. GCN Gradient Saliency Map; unchanged from the first revision, submitted; Figure S3. Cytotoxicty of latanoprost and its impurities on ocular-cell models(0.05%) by CCK8 Assay; unchanged from the first revision, submitted; Figure S4. Per-residue interaction profiles.

## Abbreviations

The following abbreviations are used in this manuscript:

Abbreviation	Full Form
ACC	Accuracy
AD	Applicability domain
AUC	Area under the receiver operating characteristic curve
BA	Balanced accuracy
CNN	Convolutional neural network
ECM	Embedded cluster modeling
FN	False negative
FP	False positive
GCN	Graph convolutional network
GHS	Globally Harmonized System
HCE-T	Human corneal epithelial cells-*trans*formed
ICH	International Council for Harmonisation
KNN	k-nearest neighbors
MCC	Matthews correlation coefficient
ML	Machine learning
OECD	Organisation for Economic Co-operation and Development
P	Precision
PBS	Phosphate-buffered saline
PGF_2α_	Prostaglandin F_2α_
pRCEC	Primary rabbit corneal epithelial cell
STR	Structure–toxicity relationship
RF	Random forest
ROC	Receiver operating characteristic
SEN	Sensitivity
SMILES	Simplified Molecular Input Line Entry System
SP	Specificity
SVM	Support vector machine
XGB	XGBoost
CCK-8	Cell Counting Kit-8
DMSO	Dimethyl sulfoxide
IC50	Half-maximal inhibitory concentration
MACCS	Molecular ACCess System
MIE	Molecular initiating event
MTT	3-(4,5-Dimethylthiazol-2-yl)-2,5-diphenyltetrazolium bromide
SLS	Sodium lauryl sulfate
STE	Short time exposure
