# Supplementary material for: Prediction of Ocular Toxicity of Prostaglandin F2α Analogs Based on Local Computational Models: Superiority over Global Models and Experimental Validation
Source: Molecules. 2026 Sep 5;31(17):3116. doi: 10.3390/molecules31173116 (PMC13566995; doi:10.3390/molecules31173116)

Supplementary Materials

Prediction of Ocular Toxicity of Prostaglandin $F_{2\alpha}$ Analogues Based on Local Computational Models: Superiority over Global Models and Experimental Validation

Manuscript ID: molecules-4489105 (Revised version)

Contents:

Table S1 350 compounds used to develop the local STR model

Table S2 SwissTargetPrediction results

Table S3 ProTox-3.0 prediction summary

Table S4 Molecular docking details

Table S5 Docking results for the four analogues and the redocked co-crystallized ligands

Figure S1 ROC curves of 16 Local STR models; unchanged from the first revision, submitted

Figure S2 GCN Gradient Saliency Map; unchanged from the first revision, submitted

Figure S3 Cytotoxicty of latanoprost and its impurities on ocular-cell models(0.05%) by CCK8 Assay; unchanged from the first revision, submitted

Figure S4 Per-residue interaction profiles

Table S1. The 350 compounds used to develop the local STR model.

SMILES	CLASS	SMILES	CLASS
C(=O)(N1c2c(C(=O)Nc3c1cccc3)csc2C)CN4CCN(CC4)C	0	C(C(Oc1ccc(C(=O)c2ccc(Cl)cc2)cc1)(C)C)(=O)O	0
O=C1N(C(=NN1CCCN2CCN(c3cc(Cl)ccc3)CC2)CC)CCOc4ccccc4	0	O=C1N(C(=O)CC1)CC	1
O=C1N(c2c(cc(Cl)cc2)N1)C3CCN(CCCN4C(=O)Nc5c4cccc5)CC3	0	O=C1C=C(CC1)C	1
C(=O)(c1ccc(NC(=O)C)cc1)NCCN(CC)CC	0	c12c(cc3c(c1)cccc3)cccc2	1
C(=O)(OCCN(CC)CC)C(c1ccccc1)c2ccccc2	0	N(=O)c1c(c2c(cc1)cccc2)O	1
C(=O)(OCCN1CCN(CCC=C2c3c(Sc4c2cccc4)ccc(Cl)c3)CC1)C	0	C(=Cc1ccccc1)(C)C	1
C(=O)(c1c(OC)cc(c(Cl)c1)N)NCCN(CC)CC	0	C(=O)(OCC1(C(=O)N(c2ccccc2)N(C1=O)c3ccccc3)CCCC)CCC(=O)O	0
N(c1ncccc1)(CCOc2ccc(cc2)CC3C(=O)NC(=O)S3)C	0	O=S(=O)(OCC12OC(OC1C3OC(OC3CO2)(C)C)(C)C)N	0
C(N(c1ccc(Cl)cc1)C2CCN(C(C)C)CC2)(=O)Cc3ccccc3	0	c1(-c2cc[n+](cc2)C)cc[n+](cc1)C	1
C(=O)(c1c(OC)cc(c(S(=O)(=O)CC)c1)N)NCC2N(CC)CCC2	0	C(=O)(OC)c1c(ccs1)N	1
C(N(c1ccccc1)C2(COC)CCN(CCN3C(=O)N(N=N3)CC)CC2)(=O)CC	0	C(=O)(O)COCC	1
O=CNc1c(O)ccc(C(O)CNC(Cc2ccc(OC)cc2)C)c1	0	O=Cc1c2c(cccc2)[nH]c1	1
N1(CCO)CCN(CCO)CC1	1	c1(NCC=C)ccccc1	1
O(c1ccc(N2CCN(C(C)C)CC2)cc1)CC3OC(c4c(Cl)cc(Cl)cc4)(OC3)Cn5ncnc5	0	Clc1cc2c(sc(n2)S)cc1	1
N(Cc1c2c(nc3C4N(C(=O)C5=C(C(C(=O)OC5)(O)CC)C=4)Cc3c2)ccc1O)(C)C	0	C(=O)(OC1C2=C(C(=O)N1c3nc4c(ccc(Cl)n4)cc3)SCCS2)N5CCN(CC5)C	0
C(=O)(C1(O)Cc2c(c3C(=O)c4c(C(=O)c3c(c2C(OC5OC(C(O)C(N)C5)C)C1)O)c(OC)ccc4)O)CO	0	C(=O)(O)CC1(CC(=O)O)CCCC1	1
C1(c2c(C=Cc3c1cccc3)cccc2)=C4CCN(CC4)C	0	C(=O)(OCC)C1C(c2ccccc2)(O1)C	1
c1(C(O)CNC(C)C)cc(O)cc(O)c1	0	c1(ccncc1)CCc2ccncc2	1
c1(ccccc1)C2OCCN(C2C)C	0	C(c1ccc(O)cc1)(c2ccc(O)cc2)(CC(C)C)C	1
c12c(Sc3c(N1CC4CN(CC4)C)cccc3)cccc2	0	N(C1CCCCC1)(C)C	1
C(NCC(O)COc1c(cccc1)C2CCCC2)(C)(C)C	0	C(=O)(F)c1ccccc1	1
O=C1N(c2ccc(N3CCN(c4ccc(OCC5CC(c6c(F)cc(F)cc6)(OC5)Cn7ncnc7)cc4)CC3)cc2)C=NN1C(C(O)C)CC	0	C(Cl)(=CCCl)C	1
C(#C)CN(C(Cc1ccccc1)C)C	0	C(=O)(C(C(=O)C)C)C	1

N(C(CN1c2c(Sc3c1cccc3)cccc2)C)(CC)CC	0	C(=O)(OC)c1c(ccc(Cl)c1)N	1
c12c(Sc3c(N1CC4C5CCN(C4)CC5)cccc3)cccc2	0	C(=O)(C1C(=O)OCC1)C	1
C(=O)(c1c(OCC(O)CNC(C)C)ccc(NC(=O)CCC)c1)C	0	Brc1c2c(c(cc1)N)cccc2	1
C(N(c1c(I)c(C(=O)NCC(O)CO)c(I)c(C(=O)NCC(O)CO)c1I)C)(=O)CO	0	C(=O)(OC)c1cc(C(=O)O)ccc1	1
C(=O)(OC(c1ccccc1)(C(CN(C)C)C)Cc2ccccc2)CC	0	O=Cc1cocc1	1
C(=O)(N1CCN(c2nc3c(c(n2)N)cc(OC)c(OC)c3)CC1)C4OCCC4	0	C(=O)(OC)c1cc(c(c(O)c1)O)O	1
C(=O)(OCCN(C)C)C	1	C(=O)(O)CC=CC	1
O=C1C(=C(N=C2N1CCCC2)C)CCN3CCC(c4c5c(on4)cc(F)cc5)CC3	0	Clc1ccc(cc1)CCO	1
N(c1ncccc1)(Cc2ccc(OC)cc2)CCN(C)C	0	O=Cc1c(O)ccc(Cl)c1	1
N(C1=NC=CC(=O)N1)(C2CCN(c3nc4c(n3Cc5ccc(F)cc5)cccc4)CC2)C	0	C(N(C)C)(=O)Cl	1
C(=O)(c1c(OC)ccc(Cl)c1)NCCc2ccc(S(=O)(=O)NC(=O)NC3CCCCC3)cc2	0	Brc1cc(c(O)cc1)CO	1
C(=O)(O)COCCN1CCN(C(c2ccc(Cl)cc2)c3ccccc3)CC1	0	OCC1C2C3C(C(C2)C1)CC(CO)C3	1
C(N(C)C)(=O)Cc1c(-c2ccc(cc2)C)nc3n1C=C(C=C3)C	0	Brc1cc2c(-c3c(cccc3)C2)cc1	1
O=C1c2c(C(=O)N1C3C(=O)NC(=O)CC3)cccc2	0	C(=O)(C=C)NCCCN(C)C	1
C(=O)(C1=C(c2c(S(=O)(=O)N1C)cccc2)O)Nc3noc(c3)C	0	N(=NNc1ccccc1)c2ccccc2	1
C(=O)(C1Oc2c(C3(C(=O)NC(=O)N3)C1)cc(F)cc2)N	0	C(=O)(OCC(CCCC)CC)c1ccc(N(C)C)cc1	1
C(=O)(c1cc(S(=O)(=O)N)c(Oc2ccccc2)c(N3CCCC3)c1)O	0	n1n[nH]cc1	1
c1(ccccc1)CN2CCC(N)CC2	1	Clc1c(c(Cl)cc(Cl)c1)NN	1
O=C1C2(OC3C(OC2OC(C1)C)C(O)C(NC)C(O)C3NC)O	0	O=Cc1c(cc(cc1C)C)C	1
O=C1C(c2ccccc2)(CC(=O)N1C)C	0	ClCC=CC	1
C(=O)(OC1CN(CC)CCC1)C(c2ccccc2)c3ccccc3	0	C(c1c(O)cc(c(Sc2c(cc(c(C(C)(C)C)c2)O)C)c1)C)(C)(C)C	1
C(c1nc(C=CC(=O)O)ccc1)(c2ccc(cc2)C)=CCN3CCCC3	0	O=S(c1nc2c(ccc(OC(F)F)c2)[nH]1)Cc3c(OC)c(OC)ccn3	0
O(c1c(OC)cc2c(c1)C3N(CC(C(CC4c5c(cc(OC)c(OC)c5)CCN4)C3)CC)CC2)C	0	c1(C=C)ccc(cc1)N	1
C(=O)(N1CCNCC1)C	1	O=C1N(Br)C(=O)CC1	1
O=C1C(c2ccc(cc2)N)(CC)CCC(=O)N1	0	C(=O)(OC1OC2C34OOC(O2)(CCC3C(CCC4C1C)C)C)CCC(=O)O	0

N(CCC=C1c2c(C=Cc3c1cccc3)cccc2)(C)C	0	C(=O)(C=C(C=CC=C(C=CC1=C(CCCC1(C)C)C)C)C)O	0
C(c1ccccc1)(c2ccccc2)(O)C3CCN(CCCC(c4ccc(C(C)(C)C)cc4)O)CC3	0	O(c1ccc(C=C)cc1)C	1
C(=O)(OCC(=O)O)Cc1c2c(n(C(=O)c3ccc(Cl)cc3)c1C)ccc(OC)c2	0	C(=O)(OC)c1oc(Br)cc1	1
C(=O)(c1c(cc(n1C)CC(=O)O)C)c2ccc(Cl)cc2	0	N(=Nc1ncc(Br)cc1)c2c(O)cc(N(CC)CC)cc2	1
C(c1ccccc1)(O)(C2C3C=CC(C2)C3)CCN4CCCCC4	0	C(=O)(O)CC(O)(C)C	1
C(=O)(OC1CC2N(C(C1)CC2)C)c3c4c(cccc4)[nH]c3	0	C(=O)(N1C(=O)CCCCC1)C	1
N(CCC=C1c2c(OCc3c1cccc3)cccc2)(C)C	0	C(=O)(c1c(c(C(C)(C)C)cc(C(C)(C)C)c1)O)O	1
N(c1cc(ccc1)C)(CCO)CC	1	C(#N)CCN(CCC#N)CCC#N	1
C(NCCO)(C)(C)C	1	C(#N)c1ccc(C(=O)N)cc1	1
c1(nn(-c2ccccc2)c(c1)C)C	1	O=C1CCCC1	1
C(=O)(C1=CC(=O)c2c(c(O)ccc2)N1)O	1	C(#N)SCCl	1
O=C1c2c(S(=O)(=O)N1)cccc2	1	C(=O)(Nc1c(Cl)cccc1)CC(=O)C	1
N(c1ccccc1)(CCO)C	1	C(c1ccc(cc1)N)(C)(C)C	1
O=C1c2c(nc[nH]2)N(C(=O)N1C)C	0	C(=O)(OCC)c1c(c2c(nc1)cccc2)O	1
C(=O)(c1c(I)c(C(=O)O)c(I)c(c1I)NC(=O)C)NCCO	0	BrC(c1ccccc1)c2ccccc2	1
c1(nnc(s1)C)N	1	C(=O)(NN)NN	1
C(=N)(NC1C(OC2OC(C(C=O)(O)C2OC3OC(C(O)C(O)C3NC)CO)C)C(O)C(O)C(NC(=N)N)C1O)N	0	C(c1cc(cc(c1)C)C)(C)(C)C	1
Fc1ccc(cc1)Cn2c3c(nc2NC4CCNCC4)cccc3	0	C(N=Nc1ccccc1)(=O)NNc2ccccc2	1
C(=O)(OCC(C(CC)C)(COC(=O)N)C)N	0	O=C1N(C(=O)C=C1)c2ccc(cc2)Cc3ccc(N4C(=O)C=CC4=O)cc3	1
C(=O)(C1OC(C(C(O)CO)O)C(NC(=O)C)C(NC(=N)N)C=1)O	0	C(=O)(c1c(Cl)cccc1)NN	1
N(c1cc(O)ccc1)(CC)CC	1	C(S)(CC)C	1
C1(NC(CC(N)C1)(C)C)(C)C	1	C(#N)C(Cl)Cl	1
C(=O)(OC)C1C(O)CCC2C1CC3c4c(c5c(cccc5)[nH]4)CCN3C2	0	N(=Nc1c(O)cccc1)c2c(O)cccc2	1
C(=O)(OCC(O)COc1c(OC)cccc1)N	0	C(#N)c1ccc(Cl)cc1	1
C(=O)(O)Cc1c(-c2ccc(Cl)cc2)cn(-c3ccc(F)cc3)n1	0	C(#C)CCC	1
S=C1c2c(N=C(N1)N)[nH]cn2	0	C(#N)c1ccc(-c2ccc(C#N)cc2)cc1	1

C(=O)(NS(=O)(=O)c1ccc(C(=O)C)cc1)NC2CCCCC2	0	C(#N)c1c(O)cccc1	1
O=C1C(=C2C(=O)c3c(cccc3)N2)Nc4c1cccc4	1	c1([n+]([O-])cccc1)S	1
C1(=NCC(c2ccccc2)O1)N	0	C(#N)CCNC	1
C(=O)(c1ccc(S(N(Cl)Cl)(=O)=O)cc1)O	1	C(#N)c1c(cccc1)C	1
Clc1nc2c(c(n1)N)ncn2C3OC(C(O)C3)CO	0	O(CCN)CCN	1
C(=O)(c1c2c(cccc2)[nH]c1)O	1	C(#N)c1c(cccc1)CCl	1
C1(c2c(cccc2)CCc3c1cccc3)=CCCNC	0	C(C(=O)CC)(=O)O	1
C(=O)(Nc1c(Cl)cccc1)C	1	C(=O)(OCCCC)c1ccccc1	1
C(=O)(Nc1cc(ccc1)C)C	1	C(#N)SCSC#N	1
N(c1cc2C(=O)OC(c2cc1)(c3ccc(N(C)C)cc3)c4ccc(N(C)C)cc4)(C)C	1	C(=NCCCC)=S	1
c1(nnnn1C)N	1	C(=O)(OCC)C=CI	1
c1(ccccc1)CNCC	1	N(N(S(=O)(=O)c1ccc(cc1)C)C)=O	1
N(CCO)C	1	O=C1C(Br)=CC(=O)C=C1	1
O=C1C=CC(I)=CN1	1	C(=CCCC(C=C)(O)C)(C)C	1
C(=O)(C=Cc1c(Cl)cccc1)O	1	C(#N)C(CC)C	1
Clc1nnnn1-c2ccccc2	1	C(=CCCC)CCC	1
c1(C(C(N)C)O)cc(O)ccc1	0	O=C1c2c(OC)c3c(c(OC)c2OC(=C1)C)occ3	1
C(=O)(c1c(O)cccc1)C=Cc2ccccc2	1	C(O)(CC(C)C)C	1
O=C1C(c2ccc(OC)cc2)=COc3c1ccc(O)c3	1	C(=O)(OCC(O)CO)c1c(Nc2c3c(c(C(F)(F)F)ccc3)ncc2)cccc1	0
c12c(c(ncn1)N)[nH]cn2	1	C(N(Cc1nc(sc1)C(C)C)C)(=O)NC(C(=O)NC(Cc2ccccc2)CC(C(NC(=O)OCc3scnc3)Cc4ccccc4)O)C(C)C	0
O=C1c2c(OC(c3cc(c(OC)cc3)O)C1)cc(O)cc2O	1	C(#N)c1cc(F)ccc1	1
c1(-c2ccncc2)nc3c(cccc3)[nH]1	1	C(=O)(O)CCCC(=O)O	1
C(O)(CNCC(O)C)C	1	C(=O)(c1ccc(N(Cc2nc3c(nc(nc3N)N)nc2)C)cc1)NC(C(=O)O)CCC(=O)O	1
O=C1C=CN=C(N1)N	1	C(CCCC)(C)C	1
C(C(c1ccccc1)(c2ccccc2)O)#C	1	C(#N)C=COCC	1

C(=O)(O)CC(O)CC(C=Cc1c(c(c(nc1C(C)C)C(C)C)COC)-c2ccc(F)cc2)O	0	C(C(C(=O)OCC)=Cc1ccccc1)#N	1
c12c(cccc1)[nH]c(n2)N	1	O=C1C(=O)C(OCC)=C1OCC	1
C(c1ccc(S(=O)(=O)Nc2c(Oc3c(OC)cccc3)c(OCCO)nc(-c4ncccn4)n2)cc1)(C)(C)C	0	O=C1NCCCCC1	1
Clc1cc2c(nc(s2)N)cc1	1	C(c1ccc(C=C)cc1)(C)(C)C	1
S(C(C)C)C(C)C	1	C(#N)c1cc(C=O)ccc1	1
C(=O)(O)COc1ccc(I)cc1	1	O(CC=C)CC(O)CO	1
C(=O)(C(CC(=O)O)CC(=O)O)O	1	O=C1C(OC(=O)N1C)(C)C	0
C(=O)(c1ccncc1)N	1	O(CCCCc1ccccc1)CCCCCCNCC(c2cc(c(O)cc2)CO)O	0
C(=O)(c1c(O)cc(O)cc1)Cc2ccccc2	1	C(=O)(c1ccccc1)C(Br)c2ccccc2	1
C(=O)(c1c(C(=O)N)cccc1)O	1	C(CO)(CO)(N)C	1
C(=O)(c1ccc(Cl)cc1)c2ccncc2	1	C(#N)Cc1ccc(O)cc1	1
Clc1c(c(Cl)ccc1)Nc2ccccc2	1	C(=O)(C=C)NC(C)(C)C	1
C(=O)(CC(C1C(=O)Oc2c(C=1O)cccc2)c3ccccc3)C	1	C(#N)Cc1cnccc1	1
C(=O)(c1c(ccc(Cl)c1)N)c2ccccc2	1	C(#N)CCOC	1
C(=C(c1ccc(O)cc1)CC)(c2ccc(O)cc2)CC	1	C(=O)(OC)C=CC(=O)OC	0
c1(-c2c(O)cccc2)nc3c(o1)cccc3	1	O=CC(CCCC)CC	1
C(#C)C(c1ccccc1)O	1	C(=O)(OCCN1C(=O)N(C(=O)N(C1=O)CCOC(=O)C=C)CCOC(=O)C=C)C=C	1
c12c(Nc3ccccc3)cccc1cccc2	1	BrCC1OCCC1	1
C(Cc1ccc(Cl)cc1)(N)(C)C	0	C(#N)C1CC=CCC1	1
O(c1cc(Nc2ccccc2)ccc1)C	1	C(#N)Cc1ccc(F)cc1	1
O(c1ccc(Nc2ccc(OC)cc2)cc1)C	1	C(=O)(OCc1ccccc1)CBr	1
O=C1NC(=O)CC(=O)N1	1	C(C(=O)CCC(=O)O)(=O)O	1
C(=O)(c1ccc(Cl)cc1)c2ccc(O)cc2	1	C(=O)(OC)c1cc(C(F)(F)F)ccc1	1
N(=Cc1ccccc1)O	1	C(=O)(CCCCC(=O)N)N	1
C(=O)(C(Oc1c(Cl)cccc1)C)O	1	C(=O)(OCC)Cc1ccccc1	1
C(=O)(c1ccc(OC)cc1)c2ccc(OC)cc2	1	O=S(=O)(c1ccccc1)C=C	1

C(=O)(O)COc1c(Cl)cccc1	1	C(=O)(OCCCCCCCCC(C)C)c1c(C(=O)OCCCCCCCC(C)C)cccc1	1
C(=O)(O)CCc1c2c(cccc2)[nH]c1	1	C(=O)(O)CCCCCc1ccccc1	1
O(c1c(OC)cccc1)CC(O)CO	0	C(=O)(OCC(COC(=O)C=C)(COC(=O)C=C)CC)C=C	1
O(c1cc2c(cc1)NC(=S)N2)C	1	C(=O)(O)CN(CC(=O)O)CCCCCCN(CC(=O)O)CC(=O)O	1
O=S(=O)(O)CCCN1CCN(CCO)CC1	1	C(=O)(c1ccccc1)CS(=O)(=O)c2ccccc2	1
c12c(oc(n1)N)cccc2	1	N(CCS(=O)(=O)O)(CCO)CCO	1
C(=O)(O)COc1c2c(ccc1)cccc2	1	C(=O)(OC(=O)CC)CC	1
C(=O)(c1cc2C(=O)c3c(C(=O)c2c(O)c1)c(O)ccc3)O	1	B(O)(O)CCC	1
C(=O)(c1ccc(F)cc1)C=Cc2ccccc2	1	C(#N)CC(=O)OCC(CCCC)CC	1
N(CCCN)(CCCC)CCCC	1	C(=O)(OCC1=C(OC(=O)O1)C)c2c3c(nc(OCC)n3Cc4ccc(-c5c(C6=NOC(=O)N6)cccc5)cc4)ccc2	0
C(=O)(OCCN1CCN(c2cc(C(F)(F)F)ccc2)CC1)c3c(Nc4c5c(ncc4)cc(C(F)(F)F)cc5)cccc3	0	C(=O)(OCCOCCOCC)C	1
C(O)(CN)CN	1	C(=O)(OCC(OCn1c2c(C(=O)N=C(N2)N)nc1)CO)C(C(C)C)N	0
c12c(oc(n1)C)ccc(-c3ccccc3)c2	1	O=S(=O)(OCC1OC1)c2cc([N+](=O)[O-])ccc2	1
C(=O)(OC)c1cc2c(cccc2)[nH]1	1	C(=O)(OC1C2(C(C(C1)CC2)(C)C)C)C=C	1
C(=O)(c1c(N=NN(C)C)[nH]cn1)N	0	C(OC)(OC)(OC)C	1
C(=O)(c1c(Cl)nccc1)O	1	C(C(C)(C)C)(=O)CBr	1
C(=O)(c1ccccc1)C(Cl)Cl	1	O(CC(O)C)CCCC	1
C(=O)(C1=CC(=O)NC(=O)N1)O	1	C(#C)CCCCO	1
C(=O)(O)Cc1c(Cl)cccc1	1	O=C1c2c(-c3c(C(=O)O1)cccc3)cccc2	1
C(=O)(OCCC)c1c(O)cccc1	1	BrCCCc1ccccc1	1
O=C1C(C2C(C(C)C)(C1)C2)C	1	C(=O)(Cl)CCC	1
C(=O)(c1ccc(cc1)N)C	1	C(=O)(Cl)C=C	1
Clc1c(c(Cl)cc(c1)N)O	1	O=S(=O)(Cl)CC	1
Clc1nc(nc(O)c1)N	1	C(=O)(C=CCCCCC)O	1
C(=O)(O)CCC1CC(CC)CC1	1	C(#N)CCCc1ccccc1	1

C(=O)(OCCC(C)C)CC(C)C	1	C(=O)(OC1CCCCC1)C=C	1
c1(c(cc(-c2ccccc2)cc1)N)O	1	C(=O)(OC)CC(=O)CCl	1
c1(-c2nc(ccc2)N)nc(ccc1)N	1	C(=CC(CC(CC(C)C)C)C)(C1C(=O)OC(=O)C1)C	1
C(=O)(c1c(-c2c(C(=O)O)cccc2)cccc1)O	1	C(=O)(c1ccc(cc1)CCCCCC)O	1
C(=O)(c1ccc(F)cc1)CC	1	N(CCCCCCCCCCCCCCCCC)(C)C	1
C(=O)(OCC)c1c(n[nH]c1)N	1	C(=O)(C(C(CO)(C)C)O)NCCC(=O)O	0
c1(-c2c(cc(cc2)N)C)c(cc(cc1)N)C	1	c1(ccc(cc1)N)CCCCCC	1
c12c(nccn1)ccc(c2)N	1	O=C1C(=C(C(=O)O1)C)C	1
c1(c(O)cccc1C)N	1	C(=C)CCCCC	1
c1(c(c(c(cc1)N)C)C)O	1	C(=O)(OCCCC)Cl	1
C(=O)(c1ccccc1)C	1	C(C(=C)C)(=O)OCCN(C)C	1
O(c1c(O)cc(cc1)CO)C	1	C(#N)CC(=O)OCC	1
c1(ccncc1)Cc2ccccc2	1	O=C1OC(=O)CC1	1
O=C1OCC2C(OC3OC4C(OC(OC4)C)C(O)C3O)c5c(cc6c(OCO6)c5)C(c7cc(OC)c(c(OC)c7)O)C12	1	O=C1OC(=O)C=C1	1
O=S(=O)(c1ccc(O)cc1)C	1	C(=O)(OCC=C)Cl	1
c1(ccc(cc1)C)CN	1	C(C(=O)Cl)(=O)OC	1
c1(-c2c3c(ccc2O)cccc3)c4c(ccc1O)cccc4	1	C(=O)(OCC)C(C(=O)OCC)CC=C	1
c1(c(-c2ccccc2)cccc1-c3ccccc3)O	1	O=P(OCC(CCCC)CC)(O)O	1
Clc1ccc(cc1)CCN	1	C(=NCCCCCCCCCCCCCCCCCC)=O	1
C(C(O)(C)C)(Cl)(Cl)Cl	1	C(C(Cl)(Cl)Cl)(=O)OC(C(Cl)(Cl)Cl)=O	1
c1(c(cccc1)Cc2ccc(O)cc2)O	1	C(=O)(O)CCCCCCCC=CCC(O)CCCCCC	1
c1(-c2ccccc2)c(cccc1)C	1	C(=O)(OC(C)(C)C)CCl	1
C(=S)(c1cc(ncc1)CC)N	0	C(C(=C)C)(=O)OCC(COC(C(=C)C)=O)(C)C	1
O=C1C(Cl)=C(C(=O)c2c1cccc2)N	1	C(C(=C)C)(=O)OCCCCCCOC(C(=C)C)=O	1
C(C(=C)C)(=O)OCCOC(=O)CC(=O)C	1	C(=O)(OC(C)(C)C)C=C	1

Note: 1 represents Toxic; 0 represents Non-toxic.

Table S2. Top 15 predicted protein targets of the four compounds from SwissTargetPrediction (Homo sapiens; ranked by prediction probability).

**Rank**	**Latanoprost**	**Latanoprost acid**	**15(S)-Latanoprost**	**trans-5,6-Latanoprost**
1	Prostaglandin F2-alpha receptor (PTGFR)	Prostaglandin F2-alpha receptor (PTGFR)	Prostaglandin F2-alpha receptor (PTGFR)	Prostaglandin F2-alpha receptor (PTGFR)
2	Leukotriene B4 receptor 1 (LTB4R)	Leukotriene B4 receptor 1 (LTB4R)	Induced myeloid leukemia cell differentiation protein Mcl-1 (MCL1)	Leukotriene B4 receptor 1 (LTB4R)
3	Induced myeloid leukemia cell differentiation protein Mcl-1 (MCL1)	Induced myeloid leukemia cell differentiation protein Mcl-1 (MCL1)	Prostaglandin E2 receptor EP3 subtype (PTGER3)	Induced myeloid leukemia cell differentiation protein Mcl-1 (MCL1)
4	Bcl-2-like protein 1 (BCL2L1)	Bcl-2-like protein 1 (BCL2L1)	Prostaglandin E2 receptor EP4 subtype (PTGER4)	Prostaglandin E2 receptor EP3 subtype (PTGER3)
5	Aromatase (CYP19A1)	Aromatase (CYP19A1)	Prostaglandin E2 receptor EP1 subtype (PTGER1)	Prostaglandin E2 receptor EP4 subtype (PTGER4)
6	Prostaglandin E2 receptor EP2 subtype (PTGER2)	Prostaglandin E2 receptor EP2 subtype (PTGER2)	Leukotriene B4 receptor 1 (LTB4R)	Prostaglandin E2 receptor EP1 subtype (PTGER1)
7	Prostaglandin E2 receptor EP3 subtype (PTGER3)	Prostaglandin E2 receptor EP3 subtype (PTGER3)	Aromatase (CYP19A1)	ATP-dependent translocase ABCB1 (ABCB1)
8	Prostaglandin E2 receptor EP4 subtype (PTGER4)	Prostaglandin E2 receptor EP4 subtype (PTGER4)	Glucocorticoid receptor (NR3C1)	Aromatase (CYP19A1)
9	Prostaglandin E2 receptor EP1 subtype (PTGER1)	Prostaglandin E2 receptor EP1 subtype (PTGER1)	Bcl-2-like protein 1 (BCL2L1)	Tyrosine-protein phosphatase non-receptor type 1 (PTPN1)

10	Nuclear receptor subfamily 5 group A member 2 (NR5A2)	Nuclear receptor subfamily 5 group A member 2 (NR5A2)	ATP-dependent translocase ABCB1 (ABCB1)	Bcl-2-like protein 1 (BCL2L1)
11	Dual specificity protein phosphatase 3 (DUSP3)	Dual specificity protein phosphatase 3 (DUSP3)	Prostaglandin G/H synthase 2 (PTGS2)	Prostaglandin E2 receptor EP2 subtype (PTGER2)
12	Glucocorticoid receptor (NR3C1)	Glucocorticoid receptor (NR3C1)	Tyrosine-protein phosphatase non-receptor type 1 (PTPN1)	Glucocorticoid receptor (NR3C1)
13	Aldo-keto reductase family 1 member C3 (AKR1C3)	Aldo-keto reductase family 1 member C3 (AKR1C3)	Prostaglandin E2 receptor EP2 subtype (PTGER2)	Tyrosine-protein phosphatase non-receptor type 2 (PTPN2)
14	Estrogen receptor (ESR1)	Estrogen receptor (ESR1)	Tyrosine-protein phosphatase non-receptor type 2 (PTPN2)	Prostaglandin G/H synthase 2 (PTGS2)
15	Estrogen receptor beta (ESR2)	Estrogen receptor beta (ESR2)	Dual specificity protein phosphatase 3 (DUSP3)	Dual specificity protein phosphatase 3 (DUSP3)

Table S3. ProTox-3.0 prediction summary for the four compounds across all 27 models. Each cell shows the call (A = active, I = inactive) and the prediction probability in parentheses. The only active prediction (NADH-quinone oxidoreductase MIE) is shown in bold.

**Category**	**Model**	**Latanoprost**	**Latanoprost acid**	**15(S)-Latanoprost**	**trans-5,6-Latanoprost**
Cytotoxicity	Cytotoxicity	I (0.75)	I (0.77)	I (0.75)	I (0.75)
Nuclear receptor	Aryl hydrocarbon receptor (AhR)	I (0.95)	I (0.95)	I (0.95)	I (0.95)
Nuclear receptor	Androgen receptor (AR)	I (0.97)	I (0.95)	I (0.97)	I (0.97)
Nuclear receptor	Androgen receptor ligand-binding domain (AR-LBD)	I (0.99)	I (0.99)	I (0.99)	I (0.99)
Nuclear receptor	Aromatase	I (0.94)	I (0.97)	I (0.94)	I (0.94)
Nuclear receptor	Estrogen receptor alpha (ER)	I (0.69)	I (0.65)	I (0.69)	I (0.69)
Nuclear receptor	Estrogen receptor ligand-binding domain (ER-LBD)	I (0.91)	I (0.94)	I (0.91)	I (0.91)
Nuclear receptor	Peroxisome proliferator-activated receptor gamma (PPAR-γ)	I (0.93)	I (0.92)	I (0.93)	I (0.93)
Stress response	Nuclear factor (erythroid-derived 2)-like 2/antioxidant responsive element (ARE)	I (0.90)	I (0.91)	I (0.90)	I (0.90)
Stress response	Heat shock factor response element (HSE)	I (0.90)	I (0.91)	I (0.90)	I (0.90)
Stress response	Mitochondrial membrane potential	I (0.80)	I (0.85)	I (0.80)	I (0.80)

	(MMP)				
Stress response	Phosphoprotein (tumor suppressor) p53	I (0.68)	I (0.93)	I (0.68)	I (0.68)
Stress response	ATPase family AAA domain-containing protein 5 (ATAD5)	I (0.97)	I (0.97)	I (0.97)	I (0.97)
MIE	Thyroid hormone receptor alpha (THRα)	I (0.53)	I (0.64)	I (0.53)	I (0.53)
MIE	Thyroid hormone receptor beta (THRβ)	I (0.71)	I (0.85)	I (0.71)	I (0.71)
MIE	Transthyretin (TTR)	I (0.66)	I (0.58)	I (0.66)	I (0.66)
MIE	Ryanodine receptor (RYR)	I (0.90)	I (0.92)	I (0.90)	I (0.90)
MIE	GABA receptor (GABAR)	I (0.72)	I (0.73)	I (0.72)	I (0.72)
MIE	Glutamate N-methyl-D-aspartate receptor (NMDAR)	I (0.99)	I (0.96)	I (0.99)	I (0.99)
MIE	alpha-amino-3-hydroxy-5-methyl-4-isoxazolepropionic acid receptor (AMPAR)	I (0.99)	I (0.99)	I (0.99)	I (0.99)
MIE	Kainate receptor (KAR)	I (1.00)	I (0.99)	I (1.00)	I (1.00)
MIE	Acetylcholinesterase (AChE)	I (0.80)	I (0.69)	I (0.80)	I (0.80)
MIE	Constitutive androstane receptor (CAR)	I (0.99)	I (0.99)	I (0.99)	I (0.99)

MIE	Pregnane X receptor (PXR)	I (0.58)	I (0.57)	I (0.58)	I (0.58)
MIE	NADH-quinone oxidoreductase (NADHOX)	**A (0.50)**	**I (0.56)**	**A (0.50)**	**A (0.50)**
MIE	Voltage-gated sodium channel (VGSC)	I (0.97)	I (0.95)	I (0.97)	I (0.97)
MIE	Na+/I- symporter (NIS)	I (0.93)	I (0.91)	I (0.93)	I (0.93)

Table S4. AutoDock Vina 1.2.7 docking parameters and complete per-pose scores for all runs (20 poses per run, fixed random seed). Redocking RMSD values (heavy atoms, best pose vs. crystallographic position): decylubiquinone 2.02 Å; amiodarone 7.1 Å.

Ligand	Target	Grid centre (x, y, z; Å)	Box (Å)	Exh./seed	Best (kcal/mol)	Top-5 mean ± SD	Within 1 kcal/mol	All 20 pose scores (kcal/mol)
Latanoprost	Complex I (6ZKC)	(72.2, 102.3, 192.7)	28 × 28 × 28	32/12345	-9.51	-9.26 ± 0.22	9/20	-9.51, -9.50, -9.24, -9.11, -8.93, -8.77, -8.72, -8.72, -8.60, -8.46, -8.37, -7.92, -7.84, -7.50, -7.47, -7.38, -7.11, -7.07, -7.04, -6.92
Latanoprost acid	Complex I (6ZKC)	(72.2, 102.3, 192.7)	28 × 28 × 28	32/12345	-10.32	-9.80 ± 0.30	5/20	-10.32, -9.97, -9.65, -9.54, -9.54, -9.29, -9.18, -9.11, -9.07, -9.00, -8.85, -8.81, -8.78, -8.73, -8.60, -8.54, -8.46, -8.22, -8.21, -8.02
15(S)-Latanoprost	Complex I (6ZKC)	(72.2, 102.3, 192.7)	28 × 28 × 28	32/12345	-10.24	-9.98 ± 0.18	9/20	-10.24, -10.12, -9.92, -9.84, -9.77, -9.50, -9.34,

								-9.27, -9.27, -9.19, -9.11, -8.89, -8.82, -8.79, -8.71, -8.55, -8.55, -8.46, -8.42, -8.02
trans-5,6-Latanoprost	Complex I (6ZKC)	(72.2, 102.3, 192.7)	28 × 28 × 28	32/12345	-10.55	-9.83 ± 0.42	4/20	-10.55, -9.99, -9.69, -9.56, -9.33, -9.27, -9.02, -8.75, -8.74, -8.60, -8.59, -8.58, -8.53, -8.43, -8.35, -8.26, -8.18, -8.11, -7.90, -7.68
Decylubiquinone (control)	Complex I (6ZKC)	(72.2, 102.3, 192.7)	28 × 28 × 28	32/12345	-7.16	-7.07 ± 0.07	20/20	-7.16, -7.13, -7.09, -6.99, -6.99, -6.97, -6.92, -6.83, -6.80, -6.80, -6.79, -6.76, -6.73, -6.62, -6.62, -6.62, -6.41, -6.40, -6.40, -6.38
Latanoprost	SIRT3 (5H4D)	(22.2, 28.1, -16.3)	26 × 26 × 26	32/12345	-6.80	-6.50 ± 0.27	10/20	-6.80, -6.65, -6.58, -6.46, -6.00, -5.92,

								-5.92, -5.90, -5.89, -5.82, -5.77, -5.75, -5.74, -5.69, -5.67, -5.62, -5.59, -5.55, -5.51, -5.41
Latanoprost acid	SIRT3 (5H4D)	(22.2, 28.1, -16.3)	26 × 26 × 26	32/12345	-6.86	-6.54 ± 0.20	15/20	-6.86, -6.67, -6.49, -6.39, -6.29, -6.28, -6.25, -6.20, -6.11, -6.10, -6.06, -6.00, -5.99, -5.97, -5.96, -5.80, -5.77, -5.73, -5.73, -5.71
15(S)-Latanoprost	SIRT3 (5H4D)	(22.2, 28.1, -16.3)	26 × 26 × 26	32/12345	-6.54	-6.34 ± 0.12	13/20	-6.54, -6.36, -6.33, -6.23, -6.21, -6.19, -6.04, -5.84, -5.76, -5.73, -5.68, -5.61, -5.56, -5.53, -5.51, -5.44, -5.42, -5.42, -5.35, -5.30
trans-5,6-Latanoprost	SIRT3 (5H4D)	(22.2, 28.1, -	26 × 26 × 26	32/12345	-7.25	-7.08 ± 0.14	12/20	-7.25, -7.23, -7.03, -6.95, -

		16.3)						6.93, -6.69, -6.65, -6.61, -6.38, -6.38, -6.33, -6.29, -6.22, -6.19, -6.19, -6.08, -6.07, -6.05, -5.96, -5.94
Amiodarone (control)	SIRT3 (5H4D)	(22.2, 28.1, -16.3)	26 × 26 × 26	32/12345	-6.02	-5.83 ± 0.23	14/20	-6.02, -6.02, -5.97, -5.69, -5.42, -5.41, -5.25, -5.21, -5.16, -5.10, -5.08, -5.07, -5.04, -5.04, -5.00, -4.97, -4.93, -4.93, -4.93, -4.93

Table S5.AutoDock Vina 1.2.7 docking results for the four analogues and the redocked co-crystallized ligands (exhaustiveness 32, 20 poses per run, fixed random seed).

Ligand	Target (PDB ID)	Best score (kcal/mol)	Top-5 mean ± SD (kcal/mol)	Poses within 1 kcal/mol	Hydrogen-bond residues (best pose)	Hydrophobic contacts (n)
Latanoprost	Complex I (6ZKC)	−9.51	−9.26 ± 0.22	9/20	NDUFS7-Ala56; ND1-Arg34	21
Latanoprost acid	Complex I (6ZKC)	−10.32	−9.80 ± 0.30	5/20	NDUFS2-Gly60, Thr156	18
15(S)-Latanoprost	Complex I (6ZKC)	−10.24	−9.98 ± 0.18	9/20	NDUFS2-Thr156; NDUFS7-Thr49, Phe50, Gly51, Met59	19
trans-5,6-Latanoprost	Complex I (6ZKC)	−10.55	−9.83 ± 0.42	4/20	NDUFS2-His59, Pro56, Tyr108	18
Decylubiquinone (control)	Complex I (6ZKC)	−7.16	−7.07 ± 0.07	20/20	NDUFS2-Gly60, Tyr108	12
Latanoprost	SIRT3 (5H4D)	−6.80	−6.50 ± 0.27	10/20	Phe157, Arg158, Tyr165, Gln169	6
Latanoprost acid	SIRT3 (5H4D)	−6.86	−6.54 ± 0.20	15/20	Phe157, Tyr165, Gln169	9
15(S)-Latanoprost	SIRT3 (5H4D)	−6.54	−6.34 ± 0.12	13/20	Phe157, Leu168, Tyr165, Gln169	9
trans-5,6-Latanoprost	SIRT3 (5H4D)	−7.25	−7.08 ± 0.14	12/20	Leu173 (weak, 3.4–3.5 Å); NAD^+ contact	11
Amiodarone (control)	SIRT3 (5H4D)	−6.02	−5.83 ± 0.23	14/20	Pro176	9

Figure S1 ROC curves of 16 Local STR models

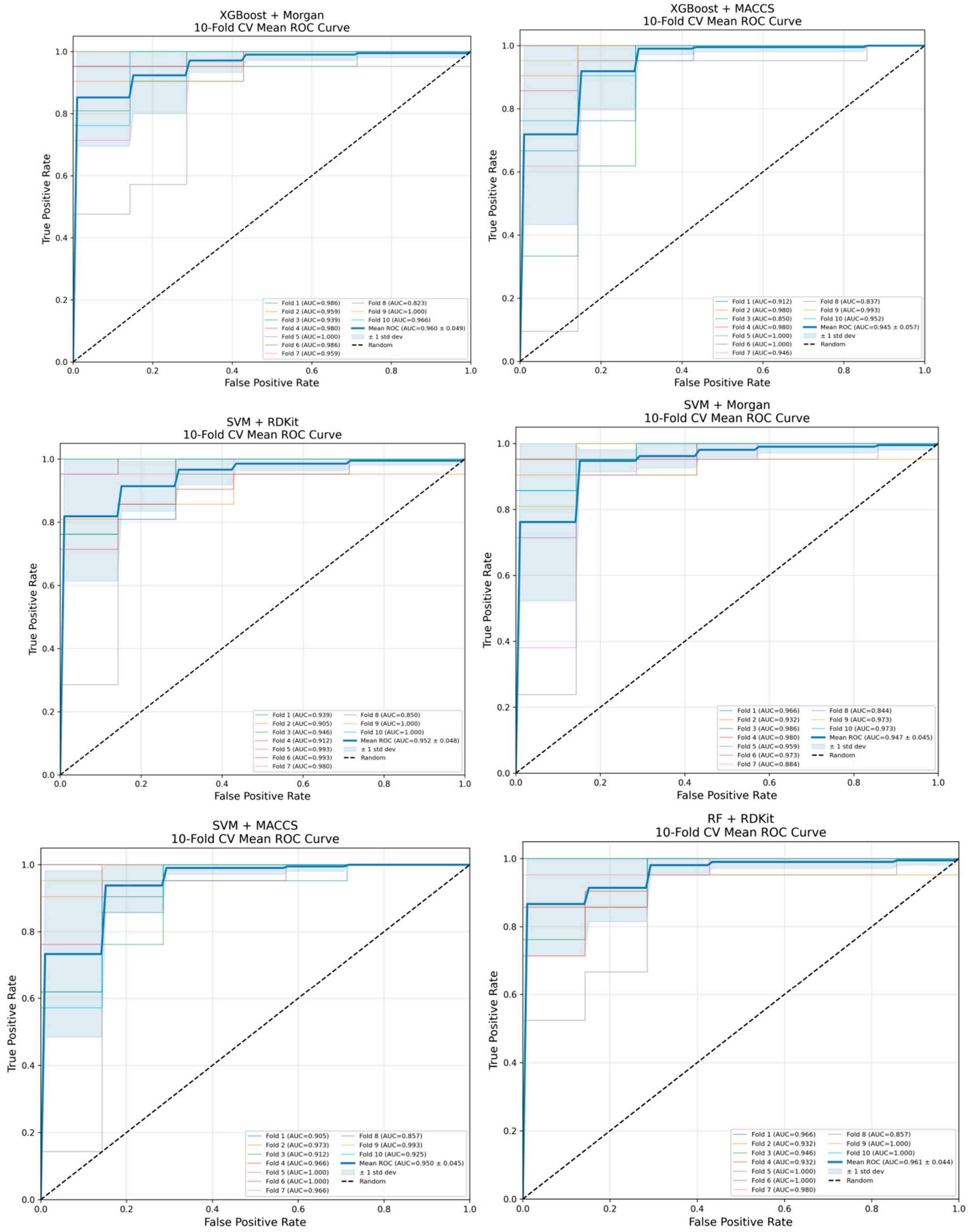

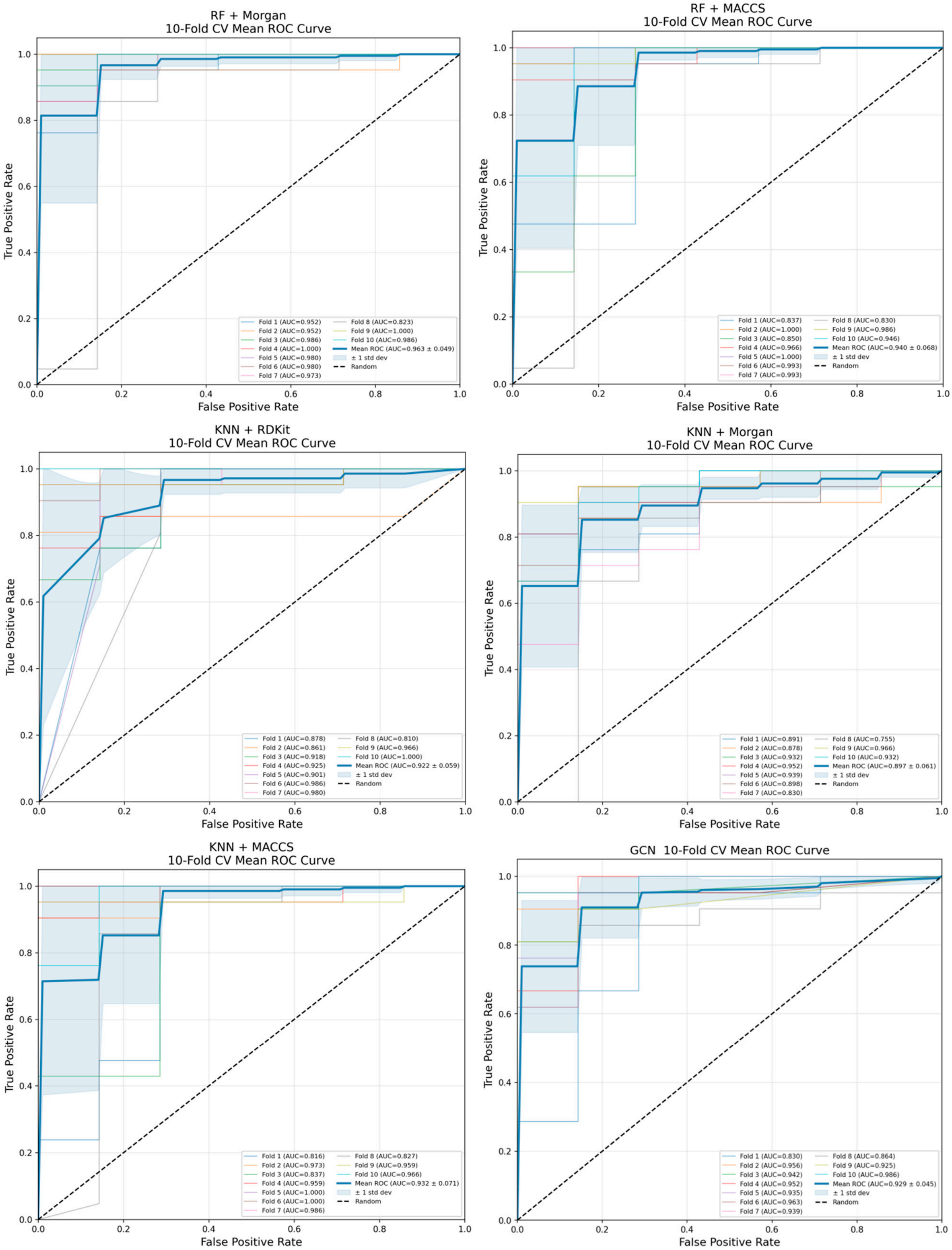
RF + Morgan
10-Fold CV Mean ROC Curve
True Positive Rate
False Positive Rate
Fold 1 (AUC=0.952)
Fold 2 (AUC=0.952)
Fold 3 (AUC=0.986)
Fold 4 (AUC=1.000)
Fold 5 (AUC=0.980)
Fold 6 (AUC=0.980)
Fold 7 (AUC=0.973)
Fold 8 (AUC=0.823)
Fold 9 (AUC=1.000)
Fold 10 (AUC=0.986)
Mean ROC (AUC=0.963 ± 0.049)
± 1 std dev
Random
RF + MACCS
10-Fold CV Mean ROC Curve
True Positive Rate
False Positive Rate
Fold 1 (AUC=0.837)
Fold 2 (AUC=1.000)
Fold 3 (AUC=0.850)
Fold 4 (AUC=0.966)
Fold 5 (AUC=1.000)
Fold 6 (AUC=0.993)
Fold 7 (AUC=0.993)
Fold 8 (AUC=0.830)
Fold 9 (AUC=0.986)
Fold 10 (AUC=0.946)
Mean ROC (AUC=0.940 ± 0.068)
± 1 std dev
Random
KNN + RDKit
10-Fold CV Mean ROC Curve
True Positive Rate
False Positive Rate
Fold 1 (AUC=0.878)
Fold 2 (AUC=0.861)
Fold 3 (AUC=0.918)
Fold 4 (AUC=0.925)
Fold 5 (AUC=0.901)
Fold 6 (AUC=0.986)
Fold 7 (AUC=0.980)
Fold 8 (AUC=0.810)
Fold 9 (AUC=0.966)
Fold 10 (AUC=1.000)
Mean ROC (AUC=0.922 ± 0.059)
± 1 std dev
Random
KNN + Morgan
10-Fold CV Mean ROC Curve
True Positive Rate
False Positive Rate
Fold 1 (AUC=0.891)
Fold 2 (AUC=0.878)
Fold 3 (AUC=0.932)
Fold 4 (AUC=0.952)
Fold 5 (AUC=0.939)
Fold 6 (AUC=0.898)
Fold 7 (AUC=0.830)
Fold 8 (AUC=0.755)
Fold 9 (AUC=0.966)
Fold 10 (AUC=0.932)
Mean ROC (AUC=0.897 ± 0.061)
± 1 std dev
Random
KNN + MACCS
10-Fold CV Mean ROC Curve
True Positive Rate
False Positive Rate
Fold 1 (AUC=0.816)
Fold 2 (AUC=0.973)
Fold 3 (AUC=0.837)
Fold 4 (AUC=0.959)
Fold 5 (AUC=1.000)
Fold 6 (AUC=1.000)
Fold 7 (AUC=0.986)
Fold 8 (AUC=0.827)
Fold 9 (AUC=0.959)
Fold 10 (AUC=0.966)
Mean ROC (AUC=0.932 ± 0.071)
± 1 std dev
Random
GCN 10-Fold CV Mean ROC Curve
True Positive Rate
False Positive Rate
Fold 1 (AUC=0.830)
Fold 2 (AUC=0.956)
Fold 3 (AUC=0.942)
Fold 4 (AUC=0.952)
Fold 5 (AUC=0.935)
Fold 6 (AUC=0.963)
Fold 7 (AUC=0.939)
Fold 8 (AUC=0.864)
Fold 9 (AUC=0.925)
Fold 10 (AUC=0.986)
Mean ROC (AUC=0.929 ± 0.045)
± 1 std dev
Random

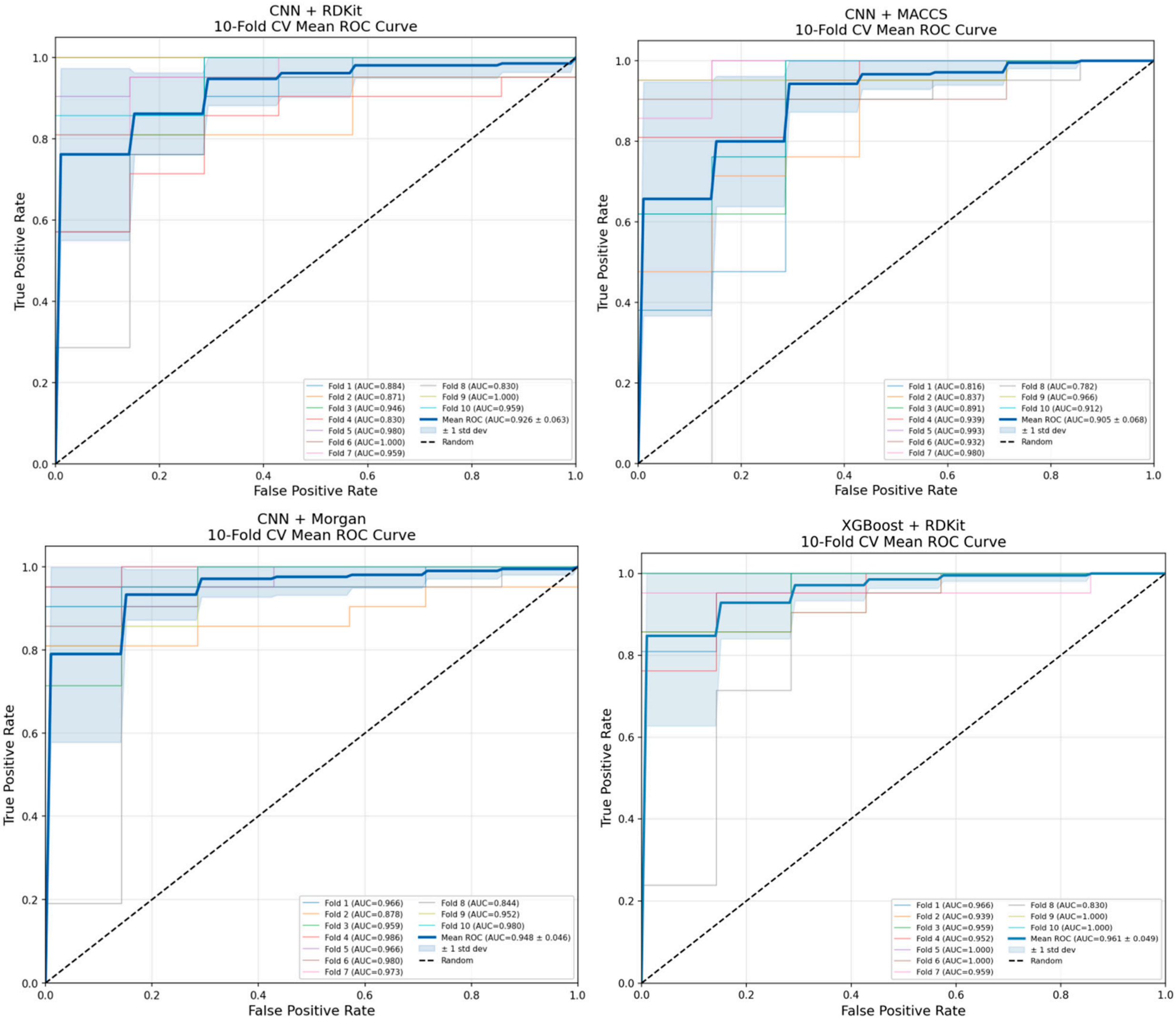
CNN + RDKit
10-Fold CV Mean ROC Curve
True Positive Rate
False Positive Rate
Fold 1 (AUC=0.884)
Fold 2 (AUC=0.871)
Fold 3 (AUC=0.946)
Fold 4 (AUC=0.830)
Fold 5 (AUC=0.980)
Fold 6 (AUC=1.000)
Fold 7 (AUC=0.959)
Fold 8 (AUC=0.830)
Fold 9 (AUC=1.000)
Fold 10 (AUC=0.959)
Mean ROC (AUC=0.926 ± 0.063)
± 1 std dev
Random
CNN + MACCS
10-Fold CV Mean ROC Curve
True Positive Rate
False Positive Rate
Fold 1 (AUC=0.816)
Fold 2 (AUC=0.837)
Fold 3 (AUC=0.891)
Fold 4 (AUC=0.939)
Fold 5 (AUC=0.993)
Fold 6 (AUC=0.932)
Fold 7 (AUC=0.980)
Fold 8 (AUC=0.782)
Fold 9 (AUC=0.966)
Fold 10 (AUC=0.912)
Mean ROC (AUC=0.905 ± 0.068)
± 1 std dev
Random
CNN + Morgan
10-Fold CV Mean ROC Curve
True Positive Rate
False Positive Rate
Fold 1 (AUC=0.966)
Fold 2 (AUC=0.878)
Fold 3 (AUC=0.959)
Fold 4 (AUC=0.986)
Fold 5 (AUC=0.966)
Fold 6 (AUC=0.980)
Fold 7 (AUC=0.973)
Fold 8 (AUC=0.844)
Fold 9 (AUC=0.952)
Fold 10 (AUC=0.980)
Mean ROC (AUC=0.948 ± 0.046)
± 1 std dev
Random
XGBoost + RDKit
10-Fold CV Mean ROC Curve
True Positive Rate
False Positive Rate
Fold 1 (AUC=0.966)
Fold 2 (AUC=0.939)
Fold 3 (AUC=0.959)
Fold 4 (AUC=0.952)
Fold 5 (AUC=1.000)
Fold 6 (AUC=1.000)
Fold 7 (AUC=0.959)
Fold 8 (AUC=0.830)
Fold 9 (AUC=1.000)
Fold 10 (AUC=1.000)
Mean ROC (AUC=0.961 ± 0.049)
± 1 std dev
Random

Figure S2   GCN Gradient Saliency Map.

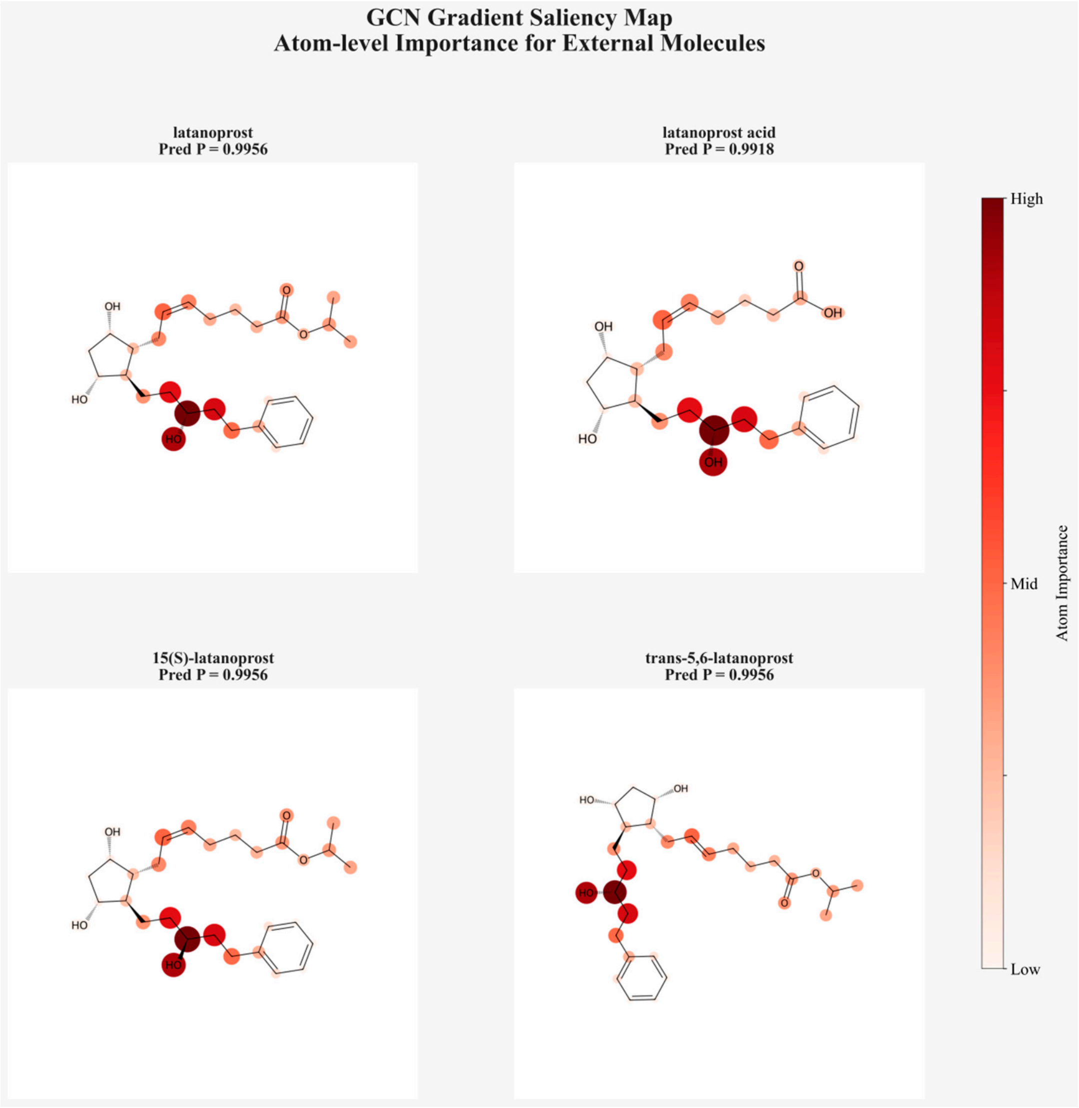

Figure S3 Cytotoxicty of latanoprost and its impurities on ocular-cell models(0.05%) by CCK8 Assay

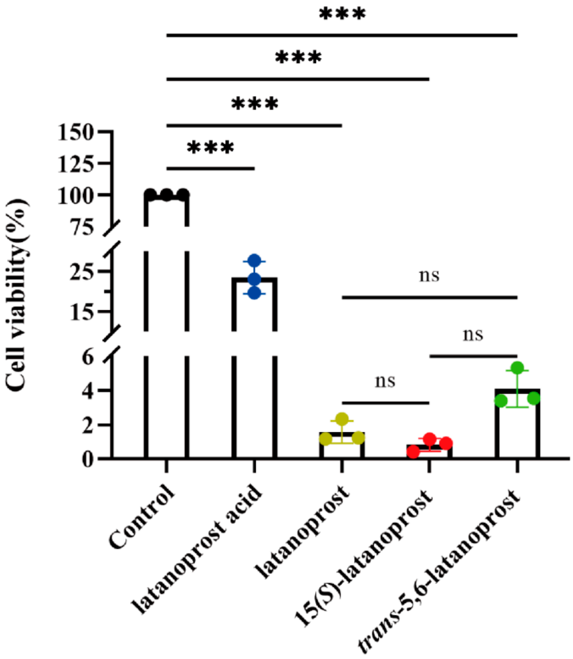

Figure S4. Per-residue interaction profiles of the best docked poses in (a) the quinone-binding channel of complex I (PDB 6ZKC) and (b) the catalytic-cleft region of SIRT3 (PDB 5H4D). Hydrogen bonds: donor–acceptor heavy-atom distance < 3.6 Å; hydrophobic contacts: carbon–carbon distance < 4.5 Å.

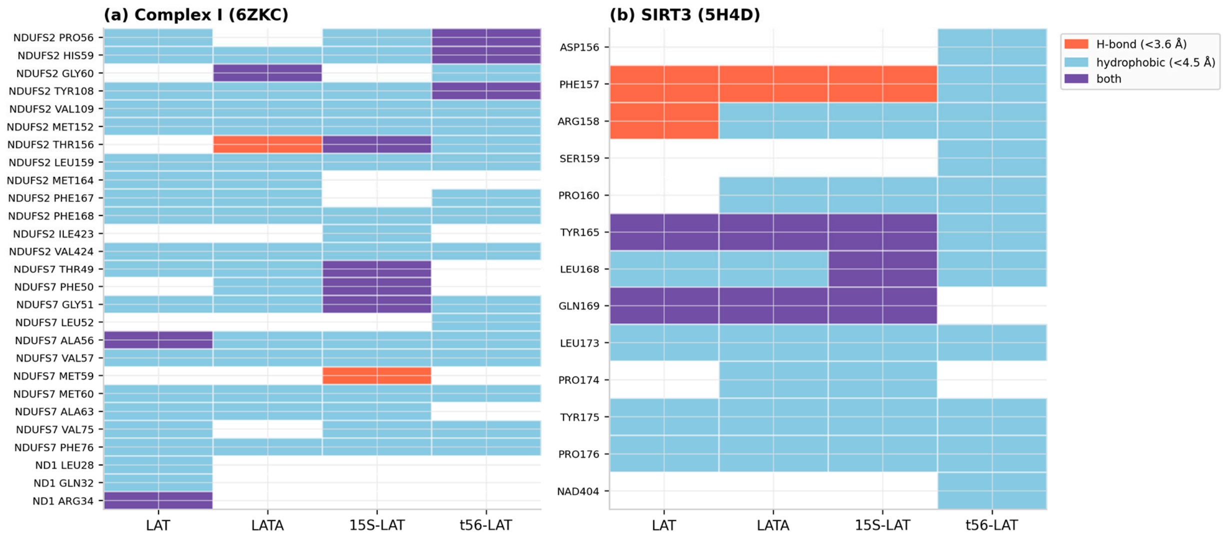

Supplement: Supplementary file 1 [file molecules-31-03116-s001.zip › molecules-4489105-supplementary.pdf]
